# Telomere-to-telomere assembly of a fish Y chromosome reveals the origin of a young sex chromosome pair

**DOI:** 10.1186/s13059-021-02430-y

**Published:** 2021-07-12

**Authors:** Lingzhan Xue, Yu Gao, Meiying Wu, Tian Tian, Haiping Fan, Yongji Huang, Zhen Huang, Dapeng Li, Luohao Xu

**Affiliations:** 1 College of Fisheries, Hubei Provincial Engineering Laboratory for Pond Aquaculture, Huazhong Agricultural University, Wuhan, 430070 China; 2 Aquaculture and Genetic Breeding Laboratory, Freshwater Fisheries Research Institute of Fujian, Fuzhou, 350002 China; 3 College of Animal Science and Technology, Key Laboratory for Plateau Fishery Resources Conservation and Sustainable Utilization of Yunnan Province, Yunnan Agricultural University, Kunming, 650201 China; 4 Freshwater Fisheries Research Institute of Fujian, Fuzhou, 350002 China; 5 Institute of Oceanography, Minjiang University, Fuzhou, 350108 China; 6 Fujian Key Laboratory of Developmental and Neural Biology & Southern Center for Biomedical Research, College of Life Sciences, Fujian Normal University, Fuzhou, Fujian China; 7 Fujian Key Laboratory of Special Marine Bio-resources Sustainable Utilization, Fuzhou, 350117 Fujian China; 8 Freshwater Aquaculture Collaborative Innovation Center of Hubei Province, Wuhan, 430070 China; 9 Department of Neurosciences and Developmental Biology, University of Vienna, 1090 Vienna, Austria

**Keywords:** Heterochromatin, Centromere, Sex chromosome, Fish genome, Recombination suppression

## Abstract

**Background:**

The origin of sex chromosomes requires the establishment of recombination suppression between the proto-sex chromosomes. In many fish species, the sex chromosome pair is homomorphic with a recent origin, providing species for studying how and why recombination suppression evolved in the initial stages of sex chromosome differentiation, but this requires accurate sequence assembly of the X and Y (or Z and W) chromosomes, which may be difficult if they are recently diverged.

**Results:**

Here we produce a haplotype-resolved genome assembly of zig-zag eel (*Mastacembelus armatus*), an aquaculture fish, at the chromosomal scale. The diploid assembly is nearly gap-free, and in most chromosomes, we resolve the centromeric and subtelomeric heterochromatic sequences. In particular, the Y chromosome, including its highly repetitive short arm, has zero gaps. Using resequencing data, we identify a ~7 Mb fully sex-linked region (SLR), spanning the sex chromosome centromere and almost entirely embedded in the pericentromeric heterochromatin. The SLRs on the X and Y chromosomes are almost identical in sequence and gene content, but both are repetitive and heterochromatic, consistent with zero or low recombination. We further identify an HMG-domain containing gene *HMGN6* in the SLR as a candidate sex-determining gene that is expressed at the onset of testis development.

**Conclusions:**

Our study supports the idea that preexisting regions of low recombination, such as pericentromeric regions, can give rise to SLR in the absence of structural variations between the proto-sex chromosomes.

**Supplementary Information:**

The online version contains supplementary material available at 10.1186/s13059-021-02430-y.

## Background

The sex chromosomes evolve from an ordinary autosome pair. Theoretical studies predict that recombination suppression in the sex-linked region (SLR) is selected when sexually antagonistic polymorphism establishes close to a sex-determining locus [1, 2]. The initial SLR can be very small and can remain so over a long time [3], but across vertebrate species, the SLR often is often large, and sometimes includes almost the entire chromosomes [4]. The differentiated sex chromosome pairs have been reported in both male heterogametic (male XY, female XX) and female heterogametic (female ZW, male ZZ) sex systems [5–7]. In eutherian mammals and neognathous birds the sex-limited chromosomes (Y or W) are gene-poor, highly repetitive and heterochromatic [8–11]. This degeneration process seems to be an inevitable consequence of recombination suppression [12–14].

In other taxa, including non-avian reptiles, amphibians, and fish, the sex chromosomes are often homomorphic [7, 15, 16]. Some homomorphic sex chromosomes have a recent origin, therefore being excellent models for studying the early stages of sex chromosome differentiation [17]. However, it has been a challenge to assemble the Y (or W) chromosome sequence with little divergence from the X (or Z). This is in part because the Y chromosome linked region may be repetitive and heterochromatic, making it difficult to sequence and assemble by short-read methods [18, 19]. PacBio CLR and Nanopore technologies produce long reads, but their error-prone nature makes it difficult to correctly differentiate the X and Y, or Z and W haplotypes [20]. Recent successes assembling vertebrate Y or W chromosomes by using long reads are limited to pairs with an intermediate [21, 22] or high [23, 24] degree of sex chromosome differentiation. When parental and progeny samples are available, the trio-binning approach permits haplotype-resolved assembly for offspring individuals [25], generating the X and Y (or Z and W) haplotype sequences, but for many non-model organisms obtaining a trio pedigree is challenging. Alternatively, high-fidelity (HiFi) long-reads produced through circular consensus sequencing are expected to have an accuracy rate greater than 99.5% [26], provide an opportunity to assemble the diploid genome, without the need for pedigree information [27]. This new sequencing technology, combined with chromatin conformation capture data, has been successfully applied to human diploid assembly [28], demonstrating its power in accurately phasing diploid genomes and resolving complex regions such as centromeres [29].In other taxa, including non-avian reptiles, amphibians, and fish, the sex chromosomes are often homomorphic [7, 15, 16]. Some homomorphic sex chromosomes have a recent origin, therefore being excellent models for studying the early stages of sex chromosome differentiation [17]. However, it has been a challenge to assemble the Y (or W) chromosome sequence with little divergence from the X (or Z). This is in part because the Y chromosome linked region may be repetitive and heterochromatic, making it difficult to sequence and assemble by short-read methods [18, 19]. PacBio CLR and Nanopore technologies produce long reads, but their error-prone nature makes it difficult to correctly differentiate the X and Y, or Z and W haplotypes [20]. Recent successes assembling vertebrate Y or W chromosomes by using long reads are limited to pairs with an intermediate [21, 22] or high [23, 24] degree of sex chromosome differentiation. When parental and progeny samples are available, the trio-binning approach permits haplotype-resolved assembly for offspring individuals [25], generating the X and Y (or Z and W) haplotype sequences, but for many non-model organisms obtaining a trio pedigree is challenging. Alternatively, high-fidelity (HiFi) long-reads produced through circular consensus sequencing are expected to have an accuracy rate greater than 99.5% [26], provide an opportunity to assemble the diploid genome, without the need for pedigree information [27]. This new sequencing technology, combined with chromatin conformation capture data, has been successfully applied to human diploid assembly [28], demonstrating its power in accurately phasing diploid genomes and resolving complex regions such as centromeres [29].

The haplotype-resolved assembly of young sex chromosome pairs is useful for resolving early events of sex chromosome differentiation, addressing questions such as why and how recombination is suppressed, and what property makes a region prone to become a SLR [17]. To illustrate how this research framework can be applied to non-model organism, we chose to assemble the diploid genome of zig-zag eel (*Mastacembelus armatus*), an aquaculture fish, with HiFi and Hi-C data. Similar to Asian swamp eel (*Monopterus albus*) [30, 31], zig-zag eel belongs to Synbranchiformes (an order in the clade Percomorpha), has a haploid number of 24 [32], and experiences female-to-male sex changes (Xue et al., submitted). Important, a recent study identified two male-specific molecular markers, implying the existence of XY sex chromosomes [33], though cytogenetically all chromosome pairs are homomorphic [34]. This made zig-zag eel an excellent model for studying the origin of sex chromosomes and the interplay between sex chromosome expression and sex change. A chromosome-level assembly of a zig-zag eel genome (fMasArm1.2) was recently available but the sex of the sequenced individual is unknown [35], and because the haplotypes have not been resolved at the chromosome scale, it has a limited power in revealing the origin of the young sex-chromosome pair.

Here we present a haplotype-resolved genome assembly of zig-zag eel, with both haploid genomes assembled into 24 chromosome models, including an X and a Y chromosome. We mapped the SLR in a pericentromeric region which has similar gene and sequence compositions between the X and Y chromosomes. We propose that the SLR which contains a candidate sex-determining gene *HMGN6* originated from the pericentromeric region of ancestrally low recombination.

## Results

### Haplotype-resolved chromosome-level assembly

**Fig. 1:**
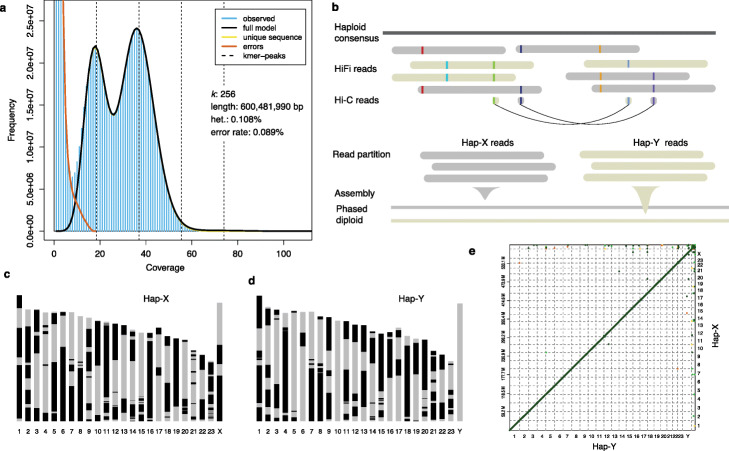
Haplotype-resolved genome assembly. **a** Estimation of the genome size and read error rate based on the distribution of k-mers (k = 256) of HiFi reads. **b** The workflow of haplotype-resolved genome assembly. The haploid consensus was used as a reference to phase the HiFi reads and Hi-C reads. The partitioned haplotype specific HiFi reads were used for separate haploid genome assembly. **c**, **d** The assembled chromosomes of both Hap-X and Hap-Y haploid. One color (black or gray) represents a continuous sequence (contig). Most chromosomes have a gap number of less than 10. **e** The genomic synteny between the Hap-X and Hap-Y genome assemblies shows that there are very few structural variations between the two haploid genomes. The unanchored scaffolds were shown at the right and top edges

We produced ~30G HiFi reads and our *k-mer* analysis suggests the error rate of HiFi reads is only 0.086% (Fig. 1a, Additional file 1: Figure S1). The long and accurate reads allow an accurate estimate of the genome size (600.1 Mb) because one can use a large *k-mer* size (256 base pairs in this case) and are useful to assemble fully phased diploid genomes. We first produced a haploid consensus contigs that were linked into chromosomes according to their anchoring positions in the existing chromosomal assembly of zig-zag eel (fMasArm1.2) [35]. The HiFi reads were then mapped against this chromosomal genome and were phased by combining the information from HiFi reads themselves and Hi-C read pairs sequenced from the same individual (Fig. 1b). On average the largest phased blocks span 99.6% of the pseudo-chromosomes, suggesting the chromosomes were able to be nearly completely phased for the two haplotypes (Additional file 2: Table S1).

The partitioned haplotype-specific HiFi reads were assembled into two haploid genomes, both having genome sizes and contig N50 values similar to those of the haploid consensus assembly (Table 1). We further extracted the haplotype-specific Hi-C read pairs and used them to link the corresponding haploid contigs into chromosomes, hence independently producing two chromosome-level haploid genomes (Fig. 1c, d). We mapped the previously identified male-specific markers [33] to only one chromosome from one haploid genome which was presumed to be the Y chromosome (corresponding to the linkage group 10 of medaka; Additional file 1: Figure S2). We therefore named the corresponding haploid genome as hap-Y and the other as hap-X. Between hap-X and hap-Y genomes, we did not detect large-scale chromosome rearrangements (Fig. 1e), but we detected a few inversions near the telomeres between our new assembly and the fMasArm1.2 assembly (Additional file 1: Figure S3). The hap-Y genome seems to have a better quality in which all of the 24 chromosomes have a gap number of less than 10, with a mean gap number of 5.5, while in the hap-X genome the mean gap number is 6.4 (Fig. 1c, d). In the following analyses, we use the hap-Y genome unless stated otherwise.

**Table 1:** The haploid and diploid genome assembly

Assembly		Size (Mb)	Contig N50 (Mb)	# contig	BUSCO completeness (%)
Haploid consensus		595.7	9.9	474	94.6
Diploid	Hap-X	582.0	7.8	364	94.4
	Hap-Y	585.6	8.6	366	97.1

Using whole-genome alignment data of seven Percomorpha species and one outgroup species Acanthochaenus luetkenii (a basal Acanthopterygian fish) [36], we confirmed that zig-zag eel is closely related to Asian swamp eel [31], and estimated they diverged from each other ~36 million years ago (Additional file 1: Figure S4).

### Karyotype evolution

**Fig. 2:**
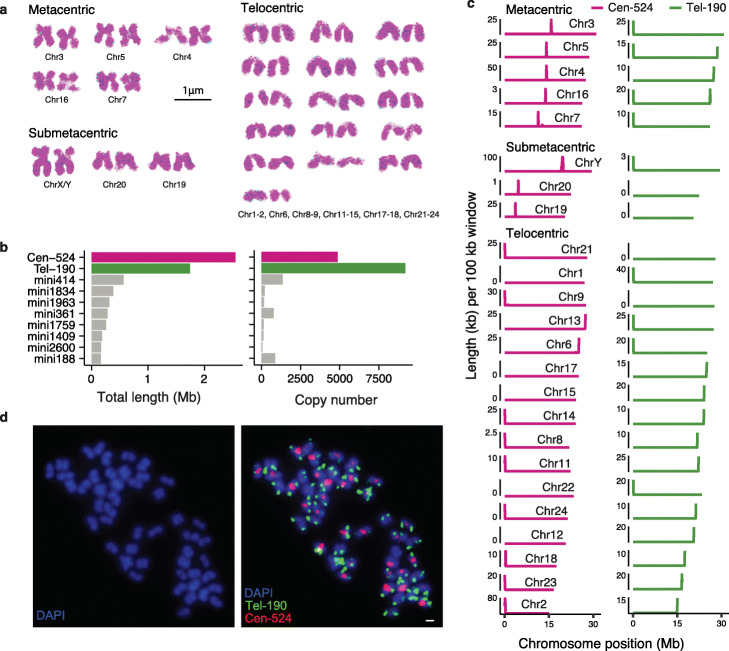
The karyotype and identification of centromeres. **a** The chromosomes were classified and grouped according to the morphology. The chromosome numbers are named according to their homology with medaka chromosomes. **b** The total length and copy number of the top 10 abundant satellite repeats. The putative centromeric (Cen-524) and telomere-associated (Tel-190) repeats were highlighted in color. The full sequences of Cen-524 and Tel-190 are available in the Additional file 8: Table S7. **c** The distribution of Cen-524 and Tel-190 satellite sequences along the chromosomes. The chromosomes were grouped according to their morphological classification in **a**. Cen-524 has only one or zero peak while in some metacentric chromosomes Tel-190 has two peaks. In a few chromosomes (e.g., Chr4) the centromeric and subtelomeric satellite sequences may have not been assembled. **d** The FISH results of Cen-524 (red) and Tel-190 (green)

Supporting the chromosome-level genome assemblies, we observed a diploid number of 48 (2n) in zig-zag eel (Fig. 2a), consistent with previous studies [32]. Based on the cytogenetic observations, we classified the chromosomes into five metacentric chromosomes, three submetacentric chromosome and 16 telocentric chromosome (Fig. 2a). Telocentric chromosomes appear to be the dominant, but larger chromosomes tend to be metacentric. To further confirm the chromosomal assembly, we analyzed the genome synteny with two additional chromosome-level genome assemblies of Percomorpha fish: Nile tilapia [37] and big-bellied seahorse. The chromosome models are highly consistent among the three genomes, despite three fusions having occurred in big-belly seahorse and two fusions in Nile tilapia (Additional file 1: Figure S5). Our synteny analyses also imply that a diploid number of 48 is an ancestral feature for those three species (Additional file 1: Figure S5), consistent with previous reports that the majority of Percomorpha fish has a diploid number of 48 [38].

### Genomic and cytogenetic identification of centromeric satellites

The high-quality genome of zig-zag eel is expected to have resolved some of the complex regions that are previously uncharacterized, such as centromeres that contains large arrays to tandem repeats [39]. To identify the centromeric satellite DNA, we searched our assembly for the most abundant satellite sequences [40] according to our repeat annotations. Two satellite sequences showed large copy numbers and had monomer lengths of 524 and 190 bp (Fig. 2b). The 524-bp satellite (named as Cen-524) usually appears at a single locus on each chromosome, and in telocentric chromosomes, it appears at one chromosomal end while in metacentric and submetacentric chromosomes it appears in the middle (Fig. 2c). This makes Cen-524 a strong candidate for the centromeric satellite. The 190-bp satellite (Tel-190), on the other hand, appears exclusively at the chromosomal ends, and on metacentric chromosomes, it is sometimes at both ends (Fig. 2c), suggesting that Tel-190 is associated with telomeres. To further validate the candidate centromeric satellite, we hybridized the probes of Cen-524 and Tel-190 using fluorescent in situ hybridization (FISH), and found that their locations on the chromosomes (Fig. 2d) are largely consistent with the genomic sequence assembly (Fig. 2c). The average size of the assembled centromeres is 50.5 kb (Additional file 3: Table S2), but we note that for most centromeres the assembly is incomplete. We were unable to assemble the conserved telomeric motif (TTAGGG)n [41] for most chromosomes, likely because the arrays of Tel-190 sequences are very long and are incomplete in our assembly, and we were unable to link the (TTAGGG)n containing contigs to the chromosomal ends. Nevertheless, our FISH experiments show the co-presence of (TTAGGG)s sequences and Tel-190 (Additional file 1: Figure S6).

### Young sex chromosome

**Fig. 3:**
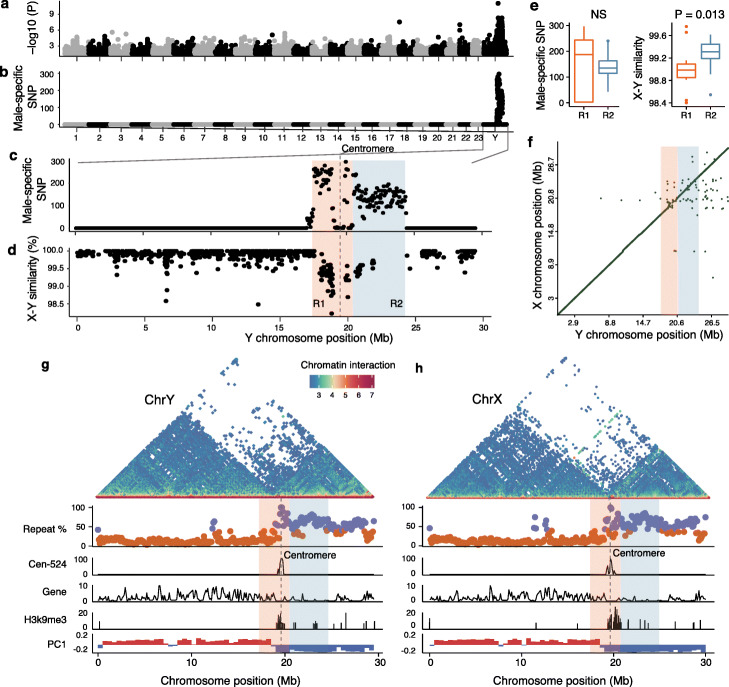
Identification of the sex-linked region. **a** *p* values (log10 transformed) of genome-wide association study for the sex trait (male or female). Re-sequencing data of ten male and ten female individuals were used. **b** The Y axis shows the density of male-specific SNPs (number per 50 kb window). Those SNPs are present in all ten males but not in females. **c** The zoom-in view of **b** on the Y chromosome. The male-specific SNPs are enriched in a ~7 Mb region. **d** The sequence similarity between the introns of the X and Y chromosomes. The similarity was calculated in every 100 kb window (black dot). Gene deserts were frequently seen in the R2 of the SLR. **e** The R1 region tend to have a higher density male-specific SNP and has a significantly lower X-Y sequence similarity than the S2 region (Wilcoxon sum rank test). **f** Sequence synteny between the X and Y chromosome. No large-scale inversions were observed. The SLR (R1 and R2) are highlighted by color. **g**, **h** Similar chromosomal compartmentalization between the X and Y chromosome. In the top panel, the colors of dots measure the frequency of chromatin interacting between 100 kb windows. Interactions across the centromere are infrequent. The first panel in the lower part shows the proportions of repeats of every 50 kb sequence (a dot) and highlighted in dark purple when it is larger than 40%, otherwise in orange. The second panel shows the proportions of the Cen-524 satellite in 100 kb windows. The third panel shows the gene density measured as the number of genes in 100 kb windows. The Y-axis of the H3K9me3 panel shows the −log 10 transformed *p* values for the H3K9me3 peak calling. The PC1 panel shows the PC1 values of Hi-C epivector: the positive values (red) represent active A compartments and the negative values (blue) represent silenced B compartments. The locations of centromeres were denoted by a dashed vertical line

As mentioned above, we used the male-specific markers to differentiate the X and Y chromosomes. To further demarcate the fully SLR, we re-sequenced 10 male and 10 female individuals and screened for variants that are associated with sex. A ~7 Mb sequence on the Y chromosome was found to be associated with sex (Fig. 3a), displaying a high density of male-specific variants (Fig. 3b, c) and increased differentiation (F_ST_) between males and females compared with the pseudoautosomal region (PAR) or autosomes (Additional file 1: Figure S7). This inferred SLR spans the centromere, and the two ends of the chromosomes are PARs. This suggests that the location physically near the centromere likely accounts for the lack of recombination of the SLR.

It seems the SLR can be divided into two regions, based on the density of male-specific variants and X-Y divergence of intronic sequences (measured with sequence similarity) (Fig. 3c, d). The first region (R1) has a significantly (Wilcoxon sum rank test, *P* = 0.013) high X-Y divergence and tends to have a higher density of male-specific SNPs than the second region (R2) (Fig. 3e). The R1 is ~3.0 Mb long, contains 67 protein-coding genes and spans the centromere (Fig. 3d), while the R2 is gene-poor, and contains only 22 genes despite its larger physical size (3.9 Mb). In both R1 and R2 the X-Y sequence divergence is close to 1% (Fig. 3d), suggesting very recent origins, and we did not detect inversions defining the two regions (Fig. 3f) or differences in gene content between the X and Y chromosomes. It is possible that R1 and R2 represent two evolutionary strata, but we cannot rule out the possibility that there is only one stratum but one region diverges faster than the other, similar to the scenario reported in emu [22].

Most of the SLR has a high density of repetitive sequences in both the X and Y chromosomes (Fig. 3g, h), and the entire short arm of the sex chromosome pair, including the centromeric regions of both the X and Y chromosomes, has features typical of heterochromatin, including an average repeat content above 50% in contrast to the long arm (~20%), a low gene density, extensive H3K9me3 modifications, and widespread inactive (B) compartments (Fig. 3g, h). Because of its physical location near the centromere, we suspect that the short-arm heterochromatin likely originated from the pericentromeric heterochromatin (PCH) [42]. The widespread heterochromatin is likely an ancestral feature that pre-dates X-Y divergence, not derived after evolution of a non-recombining Y-linked region, because it is present on both the X and Y chromosomes (Fig. 3g, h).

**Fig. 4:**
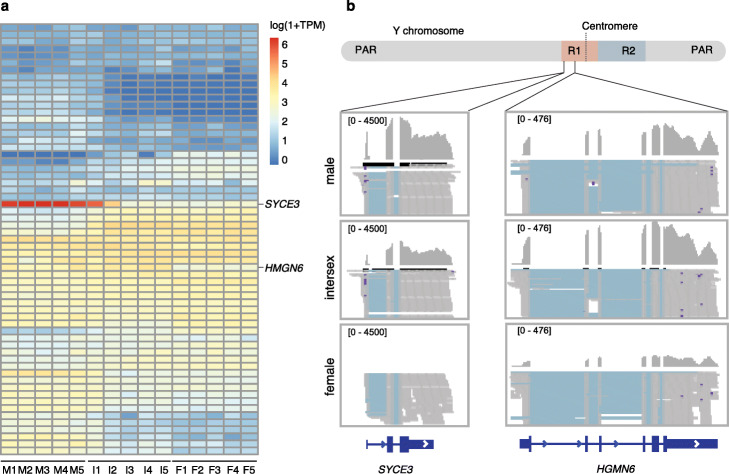
Expression of *HMGN6* and *SYCE3* in the gonads. **a** Expression profiling of the gonads of males (M), females (F), and intersex (I). RNA-seq data from five individuals (1–5) were collected for each sex category. *SYCE3* has a testis-biased expression and *HMGN6* is expressed in both testis and ovotestis. **b** The upper panel shows the Y chromosome on which the R1 (colored in salmon) and R2 (blue) locate, and the lower panel shows the alignments of gonadal RNA-seq reads from one male, one intersex, and one female for *HMGN6* and *SYCE3*

We examined the expression profiles of genes in the SLR in the gonads of males and females as well as the intersex individuals, but did not detect a tendency of tissue-specific expression (Additional file 1: Figure S8; Additional file 4: Table S3). Among the 56 expressed genes in the SLR, two attracted our attention. The first one is *SYCE3* that is exclusively expressed in testis (Additional file 1: Figure S8) but is also expressed in the late-stage of ovotestis (I2 and I4) (Fig. 4a). This suggests *SYCE3* is probably involved in spermatogenesis or other biological processes in mature testis, consistent with its known role in meiosis [43] and its high expression in Sertoli cells (Additional file 1: Figure S9). In contrast, *HMGN6* is expressed in the testis and early stages of ovotestis at a similar level, but at a much lower level in ovaries (Fig. 4a), making it a candidate gene for sex determination that directs the development of the testis. Interestingly, *HMGN6* has an HMG (High Mobility Group) domain which is also present in the mammalian master sex-determining gene *Sry*. *HMGN6* seems to be present only in teleost, but our phylogenetic and synteny analyses suggest that it is closely related to *HMGN5* (Additional file 1: Figure S10). Both *SYCE3* and *HMGN6* are located in the R1, and *SYCE3* is very close to the boundary of the SLR and the pseudoautosomal region (PAR) (Fig. 4b).

### Broad pericentromeric heterochromatin domains

**Fig. 5:**
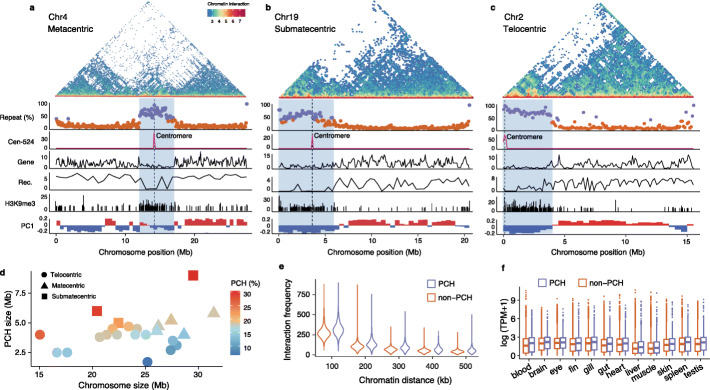
The impact of pericentromeric heterochromatin on genome compartmentalization. **a**–**c** Three chromosomes (Chr4, Chr19, Chr2) representing the metacentric, submetacentric, and telocentric chromosomes, respectively. The legends for the Hi-C contact map, repeat content, distribution of Cen-524, gene density, H3K9me3 modification, and A/B compartments are similar to these described in Fig. 3g, h. The Rec. panel shows the recombination rate (c/bp) estimated with variable window sizes based on the number of available variants. **d** Positive correlation of the chromosome size and PCH size. The colors are the measures of the proportions of PCH over the chromosomes. **e** Interaction is more frequent between PCH chromatin over various genomic distance than between non-PCH. **f** Genes are expressed at a higher level in PCH than in non-PCH

We next explored whether autosomes also have large pericentromeric regions as seen in the XY sex chromosomes. To demarcate the boundaries of pericentromeric regions, we examined repeat abundances along the chromosomes. Large regions (~4 Mb) around the centromeres have elevated repeat contents, typically higher than 50%, while the rest of the genome has less than 16% (Fig. 5a–c, Additional file 5: Table S4). These highly repetitive regions have a lower gene density, lower recombination rate (Additional file 6: Table S5) and more frequent H3k9me3 modifications (accounting for 53.7% of the genome-wide H3K9me3 peaks, versus only 17.7% of the genome, see Fig. 5a–c, Additional file 1: Figure S11-14), consistent with PCH.

The sizes of PCH are only weakly and non-significantly correlated with chromosome sizes (Pearson’s r = 0.34, *P* = 0.11). Most of them are around 4.2 Mb long (Fig. 5d), so that the smaller chromosomes, particularly the telocentric and submetacentric ones, have larger proportions of PCH (Fig. 5d, Additional file 7: Table S6). This includes the XY chromosomes (Fig. 3g, h), which are submetacentric, and almost the entire short arm of submetacentric chromosomes are heterochromatic (Additional file 1: Figure S11). Within pericentromeric regions, chromatin interactions over large physical distances are more frequent (*P* < 2.2e−16, Wilcoxon rank sum test), consistent with their higher degree of folding and compaction (Fig. 5e). Unexpectedly, we found larger proportions of genes with high expression levels and breadths in the pericentromeric regions than other regions (Fig. 5f, Additional file 1: Figure S15). This is consistent with previous suggestions that H3K9me3 play a limited role in gene repression [44–46] and the likely presence of other epigenetic modifications regulating the expression of the genes in the PCH.

## Discussion

Our haplotype-resolved assembly of zig-zag eel revealed that this species has a young Y-linked region that is almost identical to the X-linked counterpart. In contrast to the previous zig-zag eel genome assembly using error-prone CLR reads, our approach using the HiFi reads is expected to resolve repeat-rich regions such as centromeres [29]. Indeed, the chromosome carrying the male-determining allele is gap-free and contains a large repetitive pericentromeric region. Of note, this is the only gap-free chromosome in the diploid genome, and we speculate that the divergence between the X- and Y-linked region, although low, allows clearer partitioning of haploid-specific reads that helps resolve repetitive regions. The remaining gaps in the genomes are mostly tandem repeats where the long reads are difficult to assemble unambiguously [47].

Our high-quality genome also reveals that the pericentromeric regions can take up a substantial proportion (6.8 to 30.6%) of the chromosomes. How PCH impacts 3D genome organization [48] in fish is a subject of future study. Here we demonstrate that the pericentromeric region provide an alternative location for a sex-determining allele to evolve. In autosomes of similar chromosomal organization (submetacentric or metacentric) with the proto-sex chromosome, the pericentromeric regions have very low recombination rates. Because the lowly recombining pericentromeric regions are large, occupying substantial proportions of the genome, it is possible that the origin of sex-determining allele occurred in such regions by chance. We note, however, the vast majority of the pericentromeric regions is gene-poor, and the candidate sex-determining gene *HMGN6* is very close to the centromere where recombination should be very rare. We also cannot rule out the possibility that the sex-determining gene may have been translocated into the low-recombination region. This hypothesis cannot be tested without high-quality genomes of closely related species. Besides, origins of SLRs in regions with ancestrally low recombination have been recently reported in many plant species [49–51], and in papaya, the Y-linked region is also near the centromeres [52]. Consistently, theories predict that those lowly recombining regions are predisposed to evolve SLRs [17], without the costly need to create a new non-recombining region from scratch. Our study provides an example in animals, suggesting the origin of a SLR from a region of low recombination rate is not unique to plants. A similar scenario has been reported in blue tilapia in which the SLR is highly repetitive and heterochromatic on the Z chromosome [37, 53], but direct evidence supporting its origin prior to recombination suppression is currently lacking. Expanding the study to young sex chromosomes in other fish or vertebrate species with the complete diploid genome decoded will help understand the role of pericentromeric regions in sex chromosome origin and evolution.

Given that the breeding strains of zig-zag eels take 4 years to become reproductively mature (Xue et al. submitted), functional verification of our candidate sex-determining genes is challenging. Our analyses of gonadal gene expression in males, females, and intersex individuals, however, suggest *HMGN6* is a strong candidate for sex determination which is expressed at the time when half the females change into males. Though *HMGN6* has been identified only in ray-finned fishes, we provided evidence that *HMGN6* is likely an ortholog of *HMGN5* that is present in other vertebrates. *HMGN5*, like other HMGN protein, binds to nucleosomes and activates transcription [54], and is ubiquitously expressed across mammalian tissues, but at a much higher level in testis [55, 56]. Interestingly, many transcription factors related to sex determination contain an HMG domain, including *SOX9* and *SRY* [57]. However, current functional studies on *HMGN5* are limited to mammals, so further study in fish is needed.

The other gene in the SLR we focused on is *SYCE3* which is exclusively expressed in mature testis of our study species, and is one component of the synaptonemal complex, a meiosis-specific structure [58], and is also exclusively expressed in the mammalian testis [56]. Knockout of *SYCE3* in mice blocks synapsis initiation and can cause infertility [43]. These lines of evidence suggest *SYCE3* is a male-beneficial gene involved in meiosis but is likely not a sex-determining gene. It is possible that polymorphic allele controlling the expression of *SYCE3* existed in the proto-sex chromosomes, and recombination suppression is selected to maintain the linkage disequilibrium between the sexual antagonistic allele and the sex-determining locus. However, given that the pericentromeric region where *SYCE3* and *HMGN6* reside has a very low recombination rate, it is also possible that the SLR evolved without selection to suppress the recombination [3].

## Conclusions

The combination of HiFi long reads and Hi-C data allowed us to effectively generate the haplotype-resolved genome at the chromosome-scale. This diploid genome contains the sequences of subtelomeric and pericentromeric heterochromatin of most chromosomes, including the X and Y chromosomes. The fully SLR locates in the pericentromeric region, suggesting that the ancestral lowly recombining region can give rise to a SLR without the need of selection for recombination suppression.

## Methods

### Sample collection and long-read sequencing

Total DNA was extracted from muscle tissues of one male fish using a QIAamp DNA Blood Mini Kit (Qiagen), according to the manufacturer’s instructions. Single-molecule real-time circular consensus sequencing (CCS) library preparation was conducted following the recommended protocols by Pacific Bioscience. In brief, a total of 50 μg genomic DNA was sheared to ~20 kb targeted size by using Covaris g-TUBEs (Covaris). The sheared genomic DNA was examined by Agilent 2100 Bioanalyzer DNA12000 Chip (Agilent Technologies) for size distribution. Sequencing libraries were constructed using the PacBio DNA template preparation kit 2.0 (Pacific Biosciences of California, Inc., Menlo Park, CA) for HiFi sequencing on the PacBio RS II machine (Pacific Biosciences of California, Inc.) according to the manufacturer’s instructions. The constructed libraries were sequenced on one SMRT cell.

### Haplotype-resolved genome assembly

We used the peregrine (0.1.6.1) [59] assembler to assemble the HiFi reads, with default parameters. The assembled contigs were aligned to a chromosome-level assembly of zig-zag eel GCF_900324485.2 [60], and a chromosome assembly was generated by the Ragoo (1.1) [61] program. The HiFi reads were then mapped to the this chromosome assembly, using minimap2 (2.15-r905) [62] with the parameter ‘-k 19 -O 5,56 -E 4,1 -B 5 -z 400,50 -r 2k --eqx --secondary=no’. To phase the diploid, each of the Hi-C read pairs sequenced from the same individual was mapped to the genome with BWA-MEM (0.7.16a) using the default parameters. Then the alignments of both reads were paired using the script HiC_repair from the hapCUT2 (v1.2) [63] package. To partition the haplotype-specific reads, we used a pipeline described in [64]. Briefly, the HiFi-read alignments were phased with Whatshap (0.18) [65] and hapCUP2, using the phasing information from the HiFi reads themselves, as well as the Hi-C read pairs. The two-phased haplotypes were named hap-X and hap-Y, respectively. We then partitioned the reads assigned to the hap-X and hap-Y blocks, respectively. The hap-X and hap-Y derived reads were then used for haploid genome assembly. The unphased reads, despite only a small fraction of the total reads, were added to both the hap-X and hap-Y reads for genome assembly. The peregrine assembler was again used for haploid genome assembly.

### Chromosome level assembly

We combined the two haploid genomes together as a diploid reference against which we mapped the Hi-C reads pairs. Each of the Hi-C read-pair was mapped to the reference using BWA-MEM with parameters “-A 1 -B 4 -E 50 -L 0”. To assign the Hi-C read-pairs, we required each of the Hi-C read pairs to have zero mismatches with the mapped contig, and both of the read pairs were mapped to either hap-X or hap-Y contigs. The read-pairs assigned to the hap-X and hap-Y were used to do scaffolding for hap-X and hap-Y contigs, respectively, with the 3D-DNA (180114) pipeline [66, 67]. The assigned Hi-C reads were mapped to the respective haploid genome using the Juicer (1.7.6) pipeline [68]. The alignment information was used by the 3D-DNA pipeline to produce the Hi-C contact map. The Hi-C contact map was then visualized by Juicebox (1.11.08) [69] which allowed for manual curations, including correcting inversion errors and re-joining contigs that failed to be linked by 3D-DNA.

### RNA-seq

To generate RNA sequencing data for gene annotations, total RNA was isolated from the eye, brain, skin, testis, ovary, liver, spleen, kidney, intestines, muscle, blood, fin, gill, heart, and pituitary gland of a male individual using the EasyPure RNA Kit (Transgen). Sequencing libraries were generated using the NEBNext® UltraTM RNA Library Prep Kit for Illumina® (NEB, Ipswich, MA, USA), following the manufacturer’s recommendations. The cDNA libraries was used for paired-end (2 × 125 bp) sequencing on an Illumina HiSeq Xten platform by Annoroad Gene Technology Co. Ltd. (http://www.annoroad.com).

### Genome annotation

We used RepeatModeler (2.0) to predict new repeat families in the zig-zag eel genome. To annotate tandem repeats, we searched for candidate repeat units using Tandem Repeat Finder [66] with the parameter “2 7 7 80 10 20 2000 -l 6”. The results were filtered by pyTanFinder [70] which removed the redundancy of the repeat units. The predicted repeat families and tandem repeats were combined with the existing fish repeat library from Dfam (3.1) and RepBase (20170127) as the input library for repeat annotation and masking with the program RepeatMasker (4.0.7). We used the Liftoff (1.2.1) [71] program to translate the existing RefSeq gene model annotations (GCF_900324485.2) into the new assemblies of both haplotypes, with default parameters.

### Phylogenomics

We used Last (1042) [72] to align the genomes of seven species, swamp eel (Monopterus albus) [73], Nile tilapia (*Oreochromis niloticus*) [37], yellow perch (*Perca flavescens*) [74], threespine stickleback (*Gasterosteus aculeatus*) [21], yellowbelly pufferfish (*Takifugu flavidus*) [75], big-bellied seahorse (*Hippocampus abdominalis*), and pricklefish (*Acanthochaenus luetkenii*) [36], against the hap-Y genome with the option “-m100 -E0.05 -C2”. The one-to-one best alignments were retained to build 7-way multiple alignments using MULTIZ (v11.2) [76]. We then reconstructed the phylogenetic tree using IQTREE (2.0-rc1) [77] with 1000 bootstrappings. The inferred phylogeny was used for estimation of species divergence time with Beast (2.6.0). The range of 98.0–100.5 million year was set for age calibration according to the fossil records of Acanthopterygii [36].

### FISH experiment

Amplification of the centromeric (Cen-524) and telomeric (Tel-190) sequences was conducted using the primers listed in the Additional file 2: Table S7. The purified PCR products of Cen-524 and Tel-190 were labeled with Cy5-dUTP and FITC-dUTP using Nick Translation Mix (Roche, Mannheim, Germany), respectively. Nick translation was performed at 15°C for 1.5 h. These two probes were checked via agarose gel electrophoresis. The method of chromosomal preparation has been previously described [78]. Briefly, the slides were treated with 0.01% pepsin solution in 0.1 N HCl at 37°C for 1 h, and then, a 5-min wash using 2× SSC was performed. Next, they were fixed for 1 min in 4% formaldehyde in 2× SSC; rinsed three times for 3 min in 2× SSC; dehydrated with 70%, 95%, and 100% ethanol series at room temperature for 3 min each; and finally air-dried. The chromosome preparations were denatured in 70% formamide for 1 min at 70°C. The slides were dehydrated in 70%, 95%, and 100% ethanol series at −20°C, 5 min each. The 20 μl of the hybridization mix contains 50% formamide, 10 mg/mL dextran sulfate, 2× SSC, and 100 ng of each probe was heated to 95°C for 10 min and stored at 4 °C until use. Hybridization was performed for 16 h at 37°C. After hybridization, slides were washed three times for 5 min in 2× SSC, then washed again in 1× PBS at room temperature for 5 min. Slides were mounted with DAPI (Vector Laboratories, Odessa, Florida, USA). Chromosomes and FISH signals were visualized with an Olympus BX63 fluorescence microscope (Olympus, Tokyo, Japan). Images were captured on cellSens Dimension v. 1.9 and an Olympus DP80 CCD camera (Olympus, Tokyo, Japan). Images were adjusted with Adobe Photoshop v. 8.0 (Adobe, San Jose, CA, USA).

### Analyses of sex-linked regions

We collected re-sequencing data from ten males and ten females. The sex of zig-zag eel was identified through histological analysis of gonads. Once the sex was identified, we extracted DNA from the muscle tissue. A paired-end library was constructed with an insert size of 250 base pairs (bp) according to the protocol provided by the manufacturer and was sequenced on an Illumina X ten platform. The raw reads were mapped against the genome with BWA-MEN using the default parameters. After marking the duplicates, we call single-nucleotide polymorphic (SNP) sites with the GATK (4.1.4.0) joint calling pipeline. To filter the variants, we applied the parameters “QD < 2.0 || FS > 60.0 || MQRankSum < -12.5 || RedPosRankSum < -8.0 || SOR > 3.0 || MQ < 40.0”. We used SNPeff (4.3t) [79] to select the SNPs that are heterozygous in one sex but homozygous in the other sex. We further used the EMMAX (8.22) [80] pipeline to identify the SNPs that are associated with sex. The method for estimating tissue-specificity has been described in Xu and Zhou (2020) [10].

### Gene expression

The RNA-seq data of gonads from five males, five females, and five intersex individuals were produced in Xue et al. (submitted). The raw reads were mapped against the genome using HiSat2 (2.1.0) [81] with the option “-k 4”. The numbers of reads that map to transcripts were counted with featureCount (1.6.2) [82] using default parameters. The expression levels were quantified using the TPM (transcripts per million) values.

### Recombination rate estimation

We used the ReLERNN program (1.0.0) [83] method to estimate recombination rates using the individually re-sequenced genomes of 20 individuals. This method takes advantage of recurrent neural networks, instead of using the information of linkage disequilibrium, to estimate recombination landscape with a handful of re-sequenced genomes [83]. We used the filtered variants mentioned above and further removed non-biallelic variants. Since the sex chromosomes are not diploid (the ReLERNN program requires diploid genomes), they were excluded from the analyses. We simulated and trained the datasets with the default parameters. Finally, we estimated the recombination rates in non-overlapping windows whose sizes were decided by the ReLERNN program.

### Hi-C analysis

To generate the Hi-C contact matrix, we applied the “pre” function of juicebox_tools from the Juicer package. Only intra-chromosomal interactions were calculated. Then, we extracted the count values by applying the “dump” function for each chromosome, normalizing the data with the KR method. The interaction frequency was calculated with a bin size of 50 kb. For each pair-wise interaction, we required the count number larger than 10. When visualizing the chromosome-wide matrix, we further applied the log-transformation. To call compartments, we applied the “eigenvector” function of juicebox_tools with KR normalization and calculated the eigenvector at 250 kb resolution. The first principal component of Pearson’s correlation matrix based on the intrachromosomal matrix was used as the eigenvectors.

### H3K9me3 histone modification

CUT&Tag assay was performed as described previously with modifications [84]. Briefly, native nuclei were purified from frozen liver samples as previously described [85] and were washed twice gently with wash buffer (20 mM HEPES pH 7.5; 150 mM NaCl; 0.5 mM Spermidine; 1× Protease inhibitor cocktail). A 1:50 dilution of H3K9me3 (ab8898) or IgG control antibody (normal rabbit IgG: Millipore cat. no. 12-370) was used for incubation. The secondary antibody (anti-rabbit IgG antibody, goat monoclonal: Millipore AP132) was diluted to 1:100 in the dig wash buffer. DNA was purified using the phenol-chloroform-isoamyl alcohol extraction and ethanol precipitation. The libraries were amplified by mixing the DNA with 2μL of a universal i5 and uniquely barcoded i7 primer. The size distribution of libraries was determined by Agilent 4200 TapeStation analysis. Sequencing was performed in the Illumina Novaseq 6000 using 150bp paired-end following the manufacturer’s instructions. We followed the bioinformatic pipeline described in [84] to process the sequencing reads. Briefly, we used Bowtie2 (2.3.5.1) [86] to map the raw reads against the genome with the options “--local --very-sensitive-local --no-unal --no-mixed --no-discordant -I 10 -X 700”. Read redundancy was removed by the rmdup command of SAMtools (1.11) [87]. We used macs2 (2.2.7.1) [88] to call peaks and selected peaks with −log10 *p* values larger than 8.

## Supplementary Information

**Additional file 1: Figure S1.** The distribution of HiFi read lengths. The length of all HiFi reads were calculated and their distribution was shown. The vertical dashed line shows the mean length of HiFi reads. **Figure S2.** Mapping of male-specific marker on the sex chromosome. Two previously identified male-specific marker (288 bp and 4648 bp respectively) were mapped to one chromosome in the one haploid genome. We therefore named this chromosome as the Y chromosome and the haploid genome as the hap-Y. The R1 and R2 are two different regions in the sex-linked region we identified in the section “Young sex chromosome” (see Figure 3). **Figure S3.** The dot-plot between the assembly of Hap-Y and fMasArm1.2. Hap-Y is the haploid genome of zig-zag eel produced in this study the fMasArm1.2 is the genome assembly produced by Vertebrate Genome Project (VGP). The reddish colors indicate low sequence similarity while the green colors indicate high sequence similarity. **Figure S4.** Dating of species divergence in Percomorpha. Whole genome alignments were used to estimate species divergence. The estimated ages were calibrated with the fossil record at the ancestor node (Acanthopterygii). The error range shows the 95% confidence interval. **Figure S5.** Reconstruction of Percomorpha ancestral karyotype. **a-b**) The chromosome synteny between the zig-zag eel and two other fishes: Nile tilapia and big-belly seahorse. **c**) A schematic diagram shows the changes of chromosome number during the evolution of Percomorpha species. The diploid number (2n) was shown for each species. The occurrence and times of fusions were inferred based on the parsimonious principle. **Figure S6.** FISH mapping of Cen-524 and Tel-190 probes on somatic metaphase chromosomes. Clear signals of Cen-524 were detected in most of the centromeric regions, and clear signals of Tel-190 were detected in the telomeric regions of one chromosome arm on most somatic metaphase chromosomes. In most Chromosomes the conserved telomere motif (TTAGGG)n are present and sometimes are co-present with Tel-190. Scale bars = 1 μm. **Figure S7.** population differentiation between the sexes is largest in the sex-linked region. The index of population differentiation (Fst) was calculated in 50 kb windows. The peaks of Fst are enriched on the Y chromosome. In the lower panel, the zoom-in view for the Y chromosome is shown. In the sex-linked region (17-24 Mb), the Fst values are the largest. We filtered out the windows that contain less than 40 variants. **Figure S8.** The expression profile of sex-linked genes across tissues. **a)** The SLR is divided into R1 and R2. The TPM values for testis, ovary, ovotestis are the mean across five biological replicates. For other somatic tissues, the RNA-seq experiment was performed on a single individual. **b)** The genes in SLR have similar tau values (tissue specificity) compared with genes in other part of the genome. **Figure S9.** Cellular expression of *HMGN6* and *SYCE3* in ovaries and testis. O3: Cortical alveoli oocyte; O4: primary yolk oocyte; sc: spermatocytes; si: spermatid; st: seminiferous tubules; sz: spermatozoa; zp: zonal pellucida; gc: granulosa cell; stc: Sertoli cells. *HMGN6* is expressed in spermatocytes and spermatid, while *SYCE3* is mainly expressed in Sertoli cells. Both are not expressed in ovaries. **Figure S10.** The phylogeny and gene synteny of *HMGN6*. **a)** We used the protein sequences of all HMGN family members (*HMGN1*, *HMGN2*, *HMGN3*, *HMGN5*) from multiple vertebrate species to construct a phylogenetic tree, using the maximum likelihood method. The bootstrapping values are shown at the nodes. All HMGN genes are grouped by gene, and *HMGN6* or *HMGN7* is grouped with *HMGN5* of tetrapod vertebrates. **b)** The synteny of genes near *HMGN6*/*HMGN7* or *HMGN5* is shown for multiple vertebrate species. **Figure S11.** Identification of PCH on metacentric chromosomes. In the top panel, the colors of dots measure the frequency of chromatin interacting between 100 kb windows. When the repeat content of a 50 kb sequence (a dot) is larger than 40%, it is highlighted in dark purple, otherwise in orange. The portion (%) of Cen-524 satellite in 100 kb windows. The gene density is measured as the number of genes in 100 kb windows. The recombination rate (Rec.) is estimated with selected window size based on the available variants. The Y-axis of the H3K9me3 panel shows the -log 10 transformed *p*-values for the H3K9me3 peaks. The PC1 panel shows the PC1 values of Hi-C epivector: the positive values (red) represent active (A) compartments and the negative values (blue) represent silenced (B) compartments. **Figure S12.** Identification of PCH on submetacentric chromosomes. We were unable to estimate the recombination rate for the Y chromosomes using the population data. In the top panel, the colors of dots measure the frequency of chromatin interacting between 100 kb windows. When the repeat content of a 50 kb sequence (a dot) is larger than 40%, it is highlighted in dark purple, otherwise in orange. The portion (%) of Cen-524 satellite in 100 kb windows. The gene density is measured as the number of genes in 100 kb windows. The recombination rate (Rec.) is estimated with selected window size based on the available variants. The Y-axis of the H3K9me3 panel shows the -log 10 transformed *p*-values for the H3K9me3 peaks. The PC1 panel shows the PC1 values of Hi-C epivector: the positive values (red) represent active (A) compartments and the negative values (blue) represent silenced (B) compartments. **Figure S13.** Identification of PCH on small telocentric chromosomes. In the top panel, the colors of dots measure the frequency of chromatin interacting between 100 kb windows. When the repeat content of a 50 kb sequence (a dot) is larger than 40%, it is highlighted in dark purple, otherwise in orange. The portion (%) of Cen-524 satellite in 100 kb windows. The gene density is measured as the number of genes in 100 kb windows. The recombination rate (Rec.) is estimated with selected window size based on the available variants. The Y-axis of the H3K9me3 panel shows the -log 10 transformed *p*-values for the H3K9me3 peaks. The PC1 panel shows the PC1 values of Hi-C epivector: the positive values (red) represent active (A) compartments and the negative values (blue) represent silenced (B) compartments. **Figure S14.** Identification of PCH on large telocentric chromosomes. In the top panel, the colors of dots measure the frequency of chromatin interacting between 100 kb windows. When the repeat content of a 50 kb sequence (a dot) is larger than 40%, it is highlighted in dark purple, otherwise in orange. The portion (%) of Cen-524 satellite in 100 kb windows. The gene density is measured as the number of genes in 100 kb windows. The recombination rate (Rec.) is estimated with selected window size based on the available variants. The Y-axis of the H3K9me3 panel shows the -log 10 transformed *p*-values for the H3K9me3 peaks. The PC1 panel shows the PC1 values of Hi-C epivector: the positive values (red) represent active (A) compartments and the negative values (blue) represent silenced (B) compartments. **Figure S15.** PCH contains more active genes which are expressed more broadly. **a)** The tau index for genes in PCH and non-PCH regions. PCH genes are more broadly expressed than non-PCH genes (*P* = 2.279e-11, Wilcoxon rank sum test). A lower value of tau means larger breadth of expression. **b)** A larger proportion of expressed genes in PCH than in non-PCH regions. The expressed genes were defined as those with TPM (transcript per million) larger than 1.

**Additional file 2: Table S1.** Statistics of chromosome scale phasing.

**Additional file 3: Table S2.** The size and location of the assembled centromere in the hap-Y genome.

**Additional file 4: Table S3.** Expression levels of genes in the SLR.

**Additional file 5: Table S4.** Comparison of repeat contend (%) in the pericentromeric heterochromatin (PCH) and whole genome.

**Additional file 6: Table S5.** The estimated recombination rate.

**Additional file 7: Table S6.** Statistics of PCH.

**Additional file 8: Table S7.** The primers to clone Cen-524 and Tel-190 Satellite DNA.

**Additional file 9: Table S8.** Sequencing data produced from this study.

**Additional file 10.** Review history.

## Data Availability

The diploid genome assemblies are deposited at NCBI under the accession PRJNA693890 and PRJNA693891. The raw data is available from the SRA under the accession PRJNA608290 [89]. A full list of accession IDs is available in the Additional file 9: Table S8. The scripts used in this study have been reposed at Github [90] and are under the MIT license.
