# Supplementary material for: Telomere-to-telomere assembly of a fish Y chromosome reveals the origin of a young sex chromosome pair
Source: Genome Biol. 2021 Jul 12;22:203. doi: 10.1186/s13059-021-02430-y (PMC8273981; doi:10.1186/s13059-021-02430-y)
Supplement: Supplementary file 1 — Additional file 1: Figure S1. The distribution of HiFi read lengths. The length of all HiFi reads were calculated and their distribution was shown. The vertical dashed line shows the mean length of HiFi reads. Figure S2. Mapping of male-specific marker on the sex chromosome. Two previously identified male-specific marker (288 bp and 4648 bp respectively) were mapped to one chromosome in the one haploid genome. We therefore named this chromosome as the Y chromosome and the haploid genome as the hap-Y. The R1 and R2 are two different regions in the sex-linked region we identified in the section “Young sex chromosome” (see Figure 3). Figure S3. The dot-plot between the assembly of Hap-Y and fMasArm1.2. Hap-Y is the haploid genome of zig-zag eel produced in this study the fMasArm1.2 is the genome assembly produced by Vertebrate Genome Project (VGP). The reddish colors indicate low sequence similarity while the green colors indicate high sequence similarity. Figure S4. Dating of species divergence in Percomorpha. Whole genome alignments were used to estimate species divergence. The estimated ages were calibrated with the fossil record at the ancestor node (Acanthopterygii). The error range shows the 95% confidence interval. Figure S5. Reconstruction of Percomorpha ancestral karyotype. a-b) The chromosome synteny between the zig-zag eel and two other fishes: Nile tilapia and big-belly seahorse. c) A schematic diagram shows the changes of chromosome number during the evolution of Percomorpha species. The diploid number (2n) was shown for each species. The occurrence and times of fusions were inferred based on the parsimonious principle. Figure S6. FISH mapping of Cen-524 and Tel-190 probes on somatic metaphase chromosomes. Clear signals of Cen-524 were detected in most of the centromeric regions, and clear signals of Tel-190 were detected in the telomeric regions of one chromosome arm on most somatic metaphase chromosomes. In most Chromosomes the conserved telomere motif ( [file 13059_2021_2430_MOESM1_ESM.docx]

**Fig S1 The distribution of HiFi read lengths**.

The length of all HiFi reads were calculated and their distribution was shown. The vertical dashed line shows the mean length of HiFi reads.


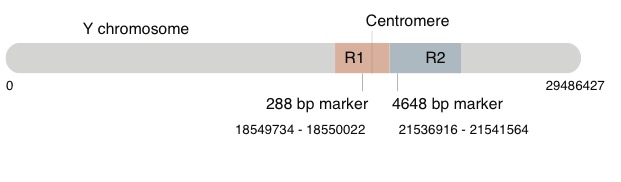


**Fig S2 Mapping of male-specific marker on the sex chromosome**

Two previously identified male-specific marker (288 bp and 4648 bp respectively) were mapped to one chromosome in the one haploid genome. We therefore named this chromosome as the Y chromosome and the haploid genome as the hap-Y. The R1 and R2 are two different regions in the sex-linked region we identified in the section “Young sex chromosome” (see Figure 3).

**Fig S3 The dot-plot between the assembly of Hap-Y and fMasArm1.2**.

Hap-Y is the haploid genome of zig-zag eel produced in this study the fMasArm1.2 is the genome assembly produced by Vertebrate Genome Project (VGP). The reddish colors indicate low sequence similarity while the green colors indicate high sequence similarity.


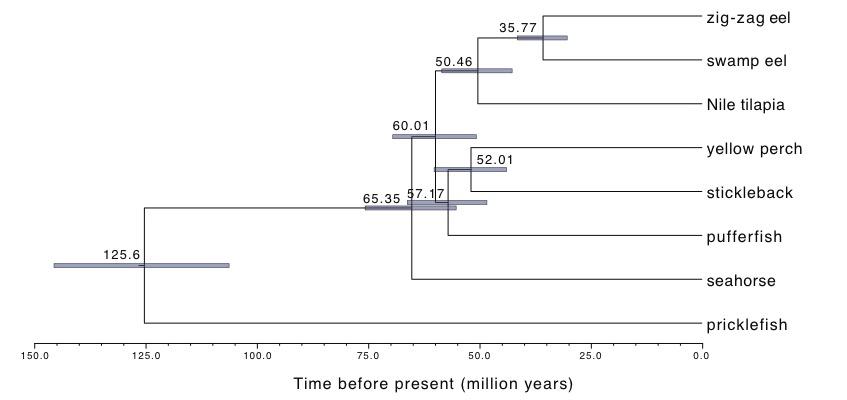


**Fig S4 Dating of species divergence in Percomorpha**.

Whole genome alignments were used to estimate species divergence. The estimated ages were calibrated with the fossil record at the ancestor node (Acanthopterygii). The error range shows the 95% confidence interval.

**Fig S5 Reconstruction of Percomorpha ancestral karyotype**.

**a-b**) The chromosome synteny between the zig-zag eel and two other fishes: Nile tilapia and big-belly seahorse. **c**) A schematic diagram shows the changes of chromosome number during the evolution of Percomorpha species. The diploid number (2n) was shown for each species. The occurrence and times of fusions were inferred based on the parsimonious principle.


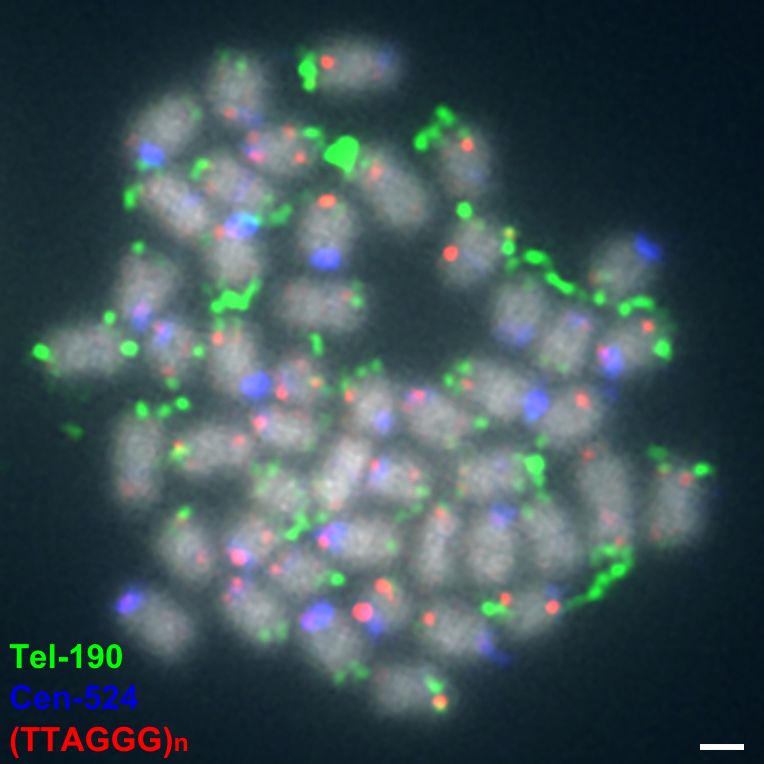


**Fig S6 FISH mapping of Cen-524 and Tel-190 probes on somatic metaphase chromosomes**.

Clear signals of Cen-524 were detected in most of the centromeric regions, and clear signals of Tel-190 were detected in the telomeric regions of one chromosome arm on most somatic metaphase chromosomes. In most Chromosomes the conserved telomere motif (TTAGGG)n are present and sometimes are co-present with Tel-190. Scale bars = 1 μm.


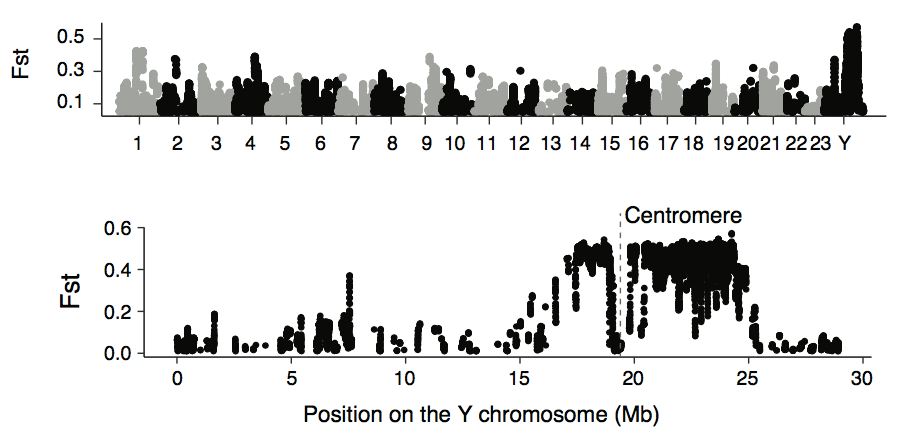


**Fig S7 population differentiation between the sexes is largest in the sex-linked region.**

The index of population differentiation (Fst) was calculated in 50 kb windows. The peaks of Fst are enriched on the Y chromosome. In the lower panel, the zoom-in view for the Y chromosome is shown. In the sex-linked region (17-24 Mb), the Fst values are the largest. We filtered out the windows that contain less than 40 variants.

**Fig S8. The expression profile of sex-linked genes across tissues**.

**a)** The SLR is divided into R1 and R2. The TPM values for testis, ovary, ovotestis are the mean across five biological replicates. For other somatic tissues, the RNA-seq experiment was performed on a single individual. **b)** The genes in SLR have similar tau values (tissue specificity) compared with genes in other part of the genome.


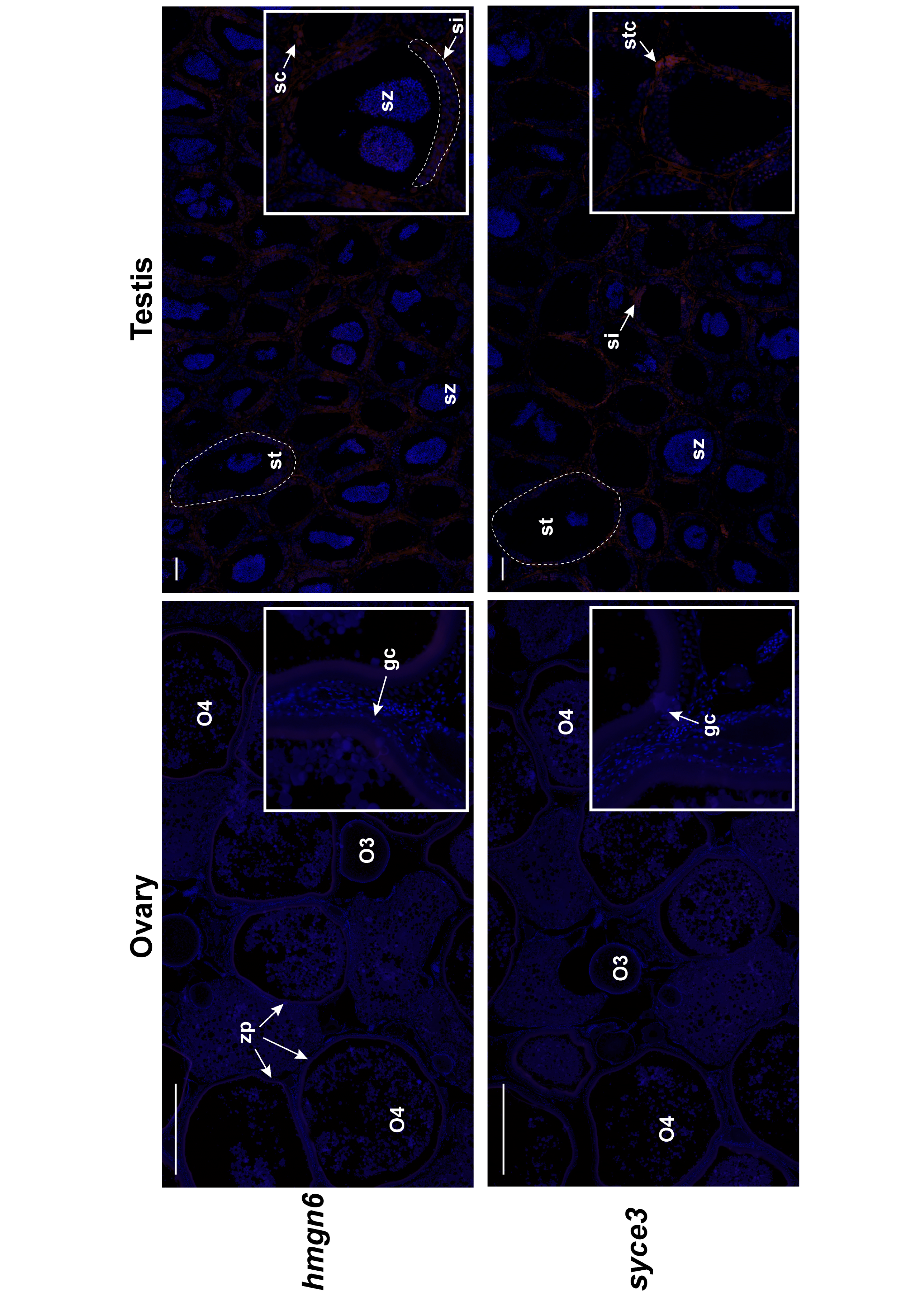


**Fig S9 Cellular expression of *HMGN6* and *SYCE3* in ovaries and testis.**

O3: Cortical alveoli oocyte; O4: primary yolk oocyte; sc: spermatocytes; si: spermatid; st: seminiferous tubules; sz: spermatozoa; zp: zonal pellucida; gc: granulosa cell; stc: Sertoli cells. *HMGN6* is expressed in spermatocytes and spermatid, while *SYCE3* is mainly expressed in Sertoli cells. Both are not expressed in ovaries.

**Fig S10 The phylogeny and gene synteny of *HMGN6*.**

**a)** We used the protein sequences of all HMGN family members (*HMGN1*, *HMGN2*, *HMGN3*, *HMGN5*) from multiple vertebrate species to construct a phylogenetic tree, using the maximum likelihood method. The bootstrapping values are shown at the nodes. All HMGN genes are grouped by gene, and *HMGN6* or *HMGN7* is grouped with *HMGN5* of tetrapod vertebrates. **b)** The synteny of genes near *HMGN6*/*HMGN7* or *HMGN5* is shown for multiple vertebrate species.

**Fig S11. Identification of PCH on metacentric chromosomes.**

In the top panel, the colors of dots measure the frequency of chromatin interacting between 100 kb windows. When the repeat content of a 50 kb sequence (a dot) is larger than 40%, it is highlighted in dark purple, otherwise in orange. The portion (%) of Cen-524 satellite in 100 kb windows. The gene density is measured as the number of genes in 100 kb windows. The recombination rate (Rec.) is estimated with selected window size based on the available variants. The Y-axis of the H3K9me3 panel shows the -log 10 transformed p-values for the H3K9me3 peaks. The PC1 panel shows the PC1 values of Hi-C epivector: the positive values (red) represent active (A) compartments and the negative values (blue) represent silenced (B) compartments.

**Fig S12. Identification of PCH on submetacentric chromosomes.**

We were unable to estimate the recombination rate for the Y chromosomes using the population data. In the top panel, the colors of dots measure the frequency of chromatin interacting between 100 kb windows. When the repeat content of a 50 kb sequence (a dot) is larger than 40%, it is highlighted in dark purple, otherwise in orange. The portion (%) of Cen-524 satellite in 100 kb windows. The gene density is measured as the number of genes in 100 kb windows. The recombination rate (Rec.) is estimated with selected window size based on the available variants. The Y-axis of the H3K9me3 panel shows the -log 10 transformed p-values for the H3K9me3 peaks. The PC1 panel shows the PC1 values of Hi-C epivector: the positive values (red) represent active (A) compartments and the negative values (blue) represent silenced (B) compartments.

**Fig S13. Identification of PCH on small telocentric chromosomes.**

In the top panel, the colors of dots measure the frequency of chromatin interacting between 100 kb windows. When the repeat content of a 50 kb sequence (a dot) is larger than 40%, it is highlighted in dark purple, otherwise in orange. The portion (%) of Cen-524 satellite in 100 kb windows. The gene density is measured as the number of genes in 100 kb windows. The recombination rate (Rec.) is estimated with selected window size based on the available variants. The Y-axis of the H3K9me3 panel shows the -log 10 transformed p-values for the H3K9me3 peaks. The PC1 panel shows the PC1 values of Hi-C epivector: the positive values (red) represent active (A) compartments and the negative values (blue) represent silenced (B) compartments.

**Fig S14. Identification of PCH on large telocentric chromosomes.**

In the top panel, the colors of dots measure the frequency of chromatin interacting between 100 kb windows. When the repeat content of a 50 kb sequence (a dot) is larger than 40%, it is highlighted in dark purple, otherwise in orange. The portion (%) of Cen-524 satellite in 100 kb windows. The gene density is measured as the number of genes in 100 kb windows. The recombination rate (Rec.) is estimated with selected window size based on the available variants. The Y-axis of the H3K9me3 panel shows the -log 10 transformed p-values for the H3K9me3 peaks. The PC1 panel shows the PC1 values of Hi-C epivector: the positive values (red) represent active (A) compartments and the negative values (blue) represent silenced (B) compartments.

**Fig S15 PCH contains more active genes which are expressed more broadly.**

**a)** The tau index for genes in PCH and non-PCH regions. PCH genes are more broadly expressed than non-PCH genes (P = 2.279e-11, Wilcoxon rank sum test). A lower value of tau means larger breadth of expression. **b)** A larger proportion of expressed genes in PCH than in non-PCH regions. The expressed genes were defined as those with TPM (transcript per million) larger than 1.
