# Supplementary material for: Telomere-to-telomere assembly of a fish Y chromosome reveals the origin of a young sex chromosome pair
Source: Genome Biol. 2021 Jul 12;22:203. doi: 10.1186/s13059-021-02430-y (PMC8273981; doi:10.1186/s13059-021-02430-y)
Supplement: Supplementary file 10 — Additional file 10. Review history. [file 13059_2021_2430_MOESM10_ESM.docx]

Review History

**First round of review**

**Reviewer 1**

**Are you able to assess all statistics in the manuscript, including the appropriateness of statistical tests used?**

Yes, and I have assessed the statistics in my report.

**Comments to author:**

General comments

This ms describes some results from a new genome sequence of a fish, probably better than previous genome assemblies. Its title should be changed as it claims that the study provides new insights, but it does not actually contribute new understanding.

One valuable aspect of the ms is that the study adds another genome assembly to those of other fish species, and these data may in the future be useful for studies of evolutionary changes in genomes, such as chromosome fusions and fissions, and other rearrangements (but the present ms does not even mention the teleost whole genome duplication until almost the end of the Discussion, and does not assess the possibility that some genes have not become diploid again, or mention how this affects the assembly of this species' genome sequence). Another is the study of the sex-determining region, which was previously located using RAD-Seq markers (though it is strange that the present manuscript fails to mention the two female specific deletions of 281 bp and 4938 bp and 156 bp male-specific deletion that were identified — are they found in the genome sequences). It is also excellent that the telomeres and centromeres have been located, and that the sequences involved were validated by in situ hybridization experiments (though these are necessarily limited to arrays of such major repeats), and by examination of the chromosome morphologies. However, it is not to make clear the numbering system. Ideally homologies with the chromosomes of other teleosts should be mentioned, and the numbering could follow that of a 'model' species, e.g. the medaka, with appropriate notation to show the claimed fusions that may have created the metacentrics, and the chromosomes involved. The sizes of the centromeric regions should also be estimated and described, perhaps in a table.

A valuable result is that the fully sex-linked region appears likely to be within a pericentromeric region. However, as explained below, the evidence that a younger evolutionary stratum, which became fully sex-linked region after the initial sex-determining region was established, is not very convincing.

Less satisfactory aspects are that the English requires very many corrections (I attach a pdf file with suggestions, as well as with comments on scientific issues), and the organization of the material is poor.

The Introduction is particularly poor. For example, it fails to explain the background about the study species of fish, and therefore does not make clear why it was chosen (i.e. what questions are being asked, and why this species is appropriate). It does not explain that the species is sequentially hermaphroditic, which might suggest that the species has an environmental sex-determination system, but the observation that 50% eventually develop into males (no reference is provided to a paper that provides this information) might instead suggest a genetic system, and this has been confirmed by finding male-specific genetic markers. As it does not have heteromorphic sex chromosomes, genome studies may be the best way to test whether it has a recently evolved, or young, system, and how its system evolved.

The Results section is also poorly organised. Also, karyotypes, and FISH experiments are mentioned but the results appeared not to be shown, and then I realised that the Figure numbers are not on the same pages as the figures, so that Figure 2 (referred to in line 179) appears to be labelled 3. Figure 4 is also referred to as 3 (also the y axes are not explained clearly). The numbering should be made clear, to avoid confusion.

Unless there are new conclusions about the mechanism of recombination suppression, the text near line 96 should be greatly shortened.

All these, and my other negative comments, can be dealt with by revisions of the manuscript, but these changes are essential, as the present version cannot be understood without a huge effort.

I have annotated the pdf file to correct the English (red text) and used purple highlighting and text to indicate scientific errors, need for more explicit information and/or references and places where the text can be shortened because it is merely saying what is well known, so that the text can simply cite a review paper, as well as repetitions.

List of major comments

The INTRODUCTION

The entire Introduction essentially says only that a fully phased and chromosome-scale assembly has been achieved of the zig-zag eel genome, a species with no detectable sex chromosome heteromorphism (references are needed for the cytological information). Several things should be in this section are not, as follows:

2. What was previously known about the relationships of the species? Do the genome data reveal for the first time that it is a close- related species of the Asian swamp eel (Monopterus albus), in the same order Synbranchiformes (line 145), or do there merely confirm this?

3. The information that most Percomorpha species have a diploid number of 48 (2n), should be mentioned in the Intro (not in line 150), along with the number of species that were studied to reach this conclusion. The intro should briefly outline what is meant by the Percomorpha.

The statement that other chromosome numbers have been observed in many taxa is obscure. If it refers to other taxa it is not relevant to what the authors are trying to say. However, it seems to refer to Percomorpha, as line 153 mentions 2 others with different numbers Nile tilapia (2n = 44) and big-bellied seahorse (2n = 42), which contradicts the statement above.

The main point is presumably that a haploid number of 24 chromosomes is expected for the species studied, and the genome assembly could be consistent with this number, and this latter point is all that needs saying the the Results section.

I do not accept that a haploid number of 24 can be inferred as ancestral for Percomorpha, as the assumptions are not explained, and alternative possibilities rejected. It is not clear why this inference is important. If it is, it should be done carefully and properly, and probably other fish should be mentioned, as 24 is a fairly common number. If fish chromosome numbers have been reviewed, the paper should be cited.

4. Very oddly, the Introduction fails to mention that the study species of fish is sequentially hermaphroditic, and that all individuals initially develop as females but half of them later develop into males. This might be expected to suggest that the species has an environmental sex-determination system, but the observation that 50% eventually develop into males (a reference to a paper that provides proper information is needed here) might instead suggest a genetic system, and this has been confirmed by RAD seq, which found male-specific genetic markers.

5. The Intro should also mention the previous chromosomal assembly of zig-zag eel (fMasArm1.2) in ref.38, and explain clearly why a new assembly is needed.

6. The Introduction should also outline HiFi sequencing, as this is a new approach and its basis, and the advantages and disadvantages, should be explained briefly.

VAGUE STATEMENTS THAT SHOULD BE MADE MUCH CLEARER.

1. Line 100 states that "Newly accumulated transposable elements (TEs) in the partially sex-linked region can also trigger the formation of a new SLR by obstructing recombination, and cites ref. [11]. However, this is not an accepted view and therefore it is necessary to explain (briefly but very clearly) how this is supposed to work. It is misleading to say that "In other taxa, the mechanism of recombination of suppression is less clear" as it is not at all clear in the birds that this reference describes, and it is inaccurate to say that "it seems sometimes the recombination is gradually ceased " (meaning "sometimes there have been several distinct recombination suppression events" — not gradual cessation).

2. The results section should describe the evidence that the genome assembly is nearly gap-free. At present, this seems to be asserted, and the evidence is not clear.

3. It is unclear what is meant by the suggestion that "highly repetitive [regions], such as centromeres, … may have important impacts on genome functions], as this statement is extremely vague (of course centromeres have important functions, but this is not worth mentioning, as it is well known. If the authors are trying to tell the reader something new, it needs to be less vague.

4. The units of y axes in Figure 5 are not always clear, and are not stated in the legend.

LIST OF CONCLUSIONS THAT ARE NOT WELL SUPPORTED

1. I do not accept the conclusion that there are 2 evolutionary strata, both with X-Y sequence divergence around 1%, as there is no test of the statistical significance of the claimed difference between the 2 regions, or even confidence intervals on the divergence values, and no correction for possible sequence variation among X-linked sequences. If there really are 2 strata, the introns from which they are inferred need to be examined carefully for indels, and the alignments described in adequate detail that readers can feel assured that they are highly reliable, as even small mis-alignments could affect divergence values. The apparently striking difference in the top figure of 3a is in terms of something called "male SNPs" that is not defined, and cannot be assessed because numbers of SNPs (which I think is what the authors mean) depend on the alignment lengths. This is why divergence per nucleotide is necessary, not just counts. The panel showing a divergence measure (mystifyingly called "similarity") show at best small differences between the two regions that the authors highlight, and no confidence intervals are shown.

If there is no definitively younger stratum, one cannot hope to "identify the evolutionary forces that favor the recombination suppression in the SLR"

2. The statement that recent studies on ratites suggested that a change of chromatin configuration and recombination rate can occur before sequence differentiation of the sex chromosomes is not convincing evidence for the idea that the presence of heterochromatin is a cause of suppressed recombination. It is well understood that suppressed recombination leads quickly to repetitive sequence accumulation (and evolution of heterochromatin) and that sequence divergence is much slower. Therefore, these observations are perfectly compatible with the order (i) recombination suppression (for whatever reason), (ii) evolution of heterochromatin, (iii) sequence divergence.

**Reviewer 2**

**Are you able to assess all statistics in the manuscript, including the appropriateness of statistical tests used?**

No, I do not feel adequately qualified to assess the statistics.

**Comments to author:**

In this manuscript the near gap free assembly of the zig-zag eel is described. Importantly in this study most centromere and subtelomere where sequenced on most chromosomes. The main focus of the paper is the analysis of the sex chromosomes. In depth sequencing of X and Y chromosomes supports the authors main conclusions that pericentromeric heterochromatin may have existed on the photo sex chromosome leading to low recombination and establishment of sexually antagonistic /sex determination genes. While the data are sound and conclusions interesting for the field I have some concerns with the presentation of the work.

Generally the paper will benefit from some English editing, I spotted a number of spelling or grammar errors some listed below.

Also the introduction has much information about sex chromosomes mostly in reptiles and mammals. There is also a lot of information about the sequencing strategy but I feel there is lack of information about the eel itself, how does it fit in the diversity of fish (in terms of genomes and sex chromosome systems in general). What is known about their sex determination and sexual development? Some of this information is provided in the results section. It was unclear to me if the male specific markers had been mapped to the putative XY?

The section on the identification and strata analysis is very interesting. As for the strata analysis and claim that they are young sex chromosomes I was wondering how this was established. I know many fish sex chromosomes are homomorphic but are they all "young" and is this because we apply assumptions from other species. In fish sex determination and sexual development is more dynamic and sex chromosomes and sex linked genes may have different functions) shaping their evolutionary trajectory in different ways.

Line 230: I would suggest to find a better title.

238: please remind the reader what PCH is.

There are a number of topics that the discussion touches on which are very interesting. I would have like more discussion on the other genes identified in this non recombining region and it they have a wide expression patter as has recently be described for mammalian sex chromosomes?

line 45/46 revise

line 56 revise sentence

131: what are the pseudo-chromosomes

165: found that first sentence a bit confusing.

271: revise sentence

The work represented in this manuscript is substantial and the in depth analysis specifically of the sex chromosomes is interesting. I felt that there was a lot of other interesting aspects that the authors did not discuss in much detail as they chose to focus on the sex chromosomes. However even on sex chromosomes there is little discussion and presentation e.g. of the genes in the sex specific region.

**Authors Response**

**Point-by-point responses to the reviewers’ comments:**

Reviewer #1

This ms describes some results from a new genome sequence of a fish, probably better than previous genome assemblies. Its title should be changed as it claims that the study provides new insights, but it does not actually contribute new understanding.

*R: we have changed the title from “Telomere-to-telomere assembly of a vertebrate Y chromosome reveals insights into the origin of young sex chromosomes” to “Telomere-to-telomere assembly of a fish Y chromosome reveals the origin of a young sex chromosome pair”.*

One valuable aspect of the ms is that the study adds another genome assembly to those of other fish species, and these data may in the future be useful for studies of evolutionary changes in genomes, such as chromosome fusions and fissions, and other rearrangements (but the present ms does not even mention the teleost whole genome duplication until almost the end of the Discussion, and does not assess the possibility that some genes have not become diploid again, or mention how this affects the assembly of this species' genome sequence). Another is the study of the sex-determining region, which was previously located using RAD-Seq markers (though it is strange that the present manuscript fails to mention the two female specific deletions of 281 bp and 4938 bp and 156 bp male-specific deletion that were identified — are they found in the genome sequences).

*R: The reviewer raised an important question about teleost whole-genome duplication. Given that the teleost-specific whole-genome duplication is an ancient event, we did not think it would have a major effect on genome assembly. And since this manuscript does not deal with the evolution of gene family, we did not pay too much attention to the fate of the duplicated genes. However, in light of this comment, we re-visited the result of the evolutionary analysis of HMGN6, aiming to determine the origin of HMGN6, using a phylogenetic method. We found HMGN6 or HMGN7 was actually closely related to HMGN5 present in other vertebrates. We have updated this result in the revised manuscript in line 716-717.*

*We had actually mapped the RAD-seq marker to our diploid genomes - that’s how we initially differentiated the hap-X and hap-Y genomes. We have now made it clear and mentioned the result in the manuscript in lines 288-290 and added a supplementary figure (Fig. S2).*

It is also excellent that the telomeres and centromeres have been located, and that the sequences involved were validated by in situ hybridization experiments (though these are necessarily limited to arrays of such major repeats), and by examination of the chromosome morphologies. However, it is not to make clear the numbering system. Ideally homologies with the chromosomes of other teleosts should be mentioned, and the numbering could follow that of a 'model' species, e.g. the medaka, with appropriate notation to show the claimed fusions that may have created the metacentrics, and the chromosomes involved. The sizes of the centromeric regions should also be estimated and described, perhaps in a table.

*R: Thanks for the positive remarks. We previously numbered the chromosomes according to the assembled sizes. Now we have changed the numbering according to their homologies with medaka chromosomes. We now explain the numbering system in the legend of Fig. 2 and also give the relationship of zig-zag eel chromosome IDs and medaka chromosome ID in the supplementary table S1 and S5. We have also added a supplementary table (table S2) describing the locations and sizes of the centromeres.*

A valuable result is that the fully sex-linked region appears likely to be within a pericentromeric region. However, as explained below, the evidence that a younger evolutionary stratum, which became fully sex-linked region after the initial sex-determining region was established, is not very convincing.

*R: thanks for pointing out this issue. in the revised manuscript we called the two strata as “two regions” since we did not find enough evidence to suggest that there are two strata.*

Less satisfactory aspects are that the English requires very many corrections (I attach a pdf file with suggestions, as well as with comments on scientific issues), and the organization of the material is poor.

*R: we have edited the text throughout the manuscript (the reviewer’s suggestions for edit are very helpful) and re-organized some parts of the results and methods parts.*

The Introduction is particularly poor. For example, it fails to explain the background about the study species of fish, and therefore does not make clear why it was chosen (i.e. what questions are being asked, and why this species is appropriate). It does not explain that the species is sequentially hermaphroditic, which might suggest that the species has an environmental sex-determination system, but the observation that 50% eventually develop into males (no reference is provided to a paper that provides this information) might instead suggest a genetic system, and this has been confirmed by finding male-specific genetic markers. As it does not have heteromorphic sex chromosomes, genome studies may be the best way to test whether it has a recently evolved, or young, system, and how its system evolved.

*R: we have rewritten the second half of the Introduction. We explained that we chose this species because we recently understood that it has a young sex chromosome pair. We have another manuscript submitted mainly describing the details of sex changes (focusing on the morphological, physiological and histological analyses) of this species. We added this reference (Xue et al. submitted) in the revised text.*

The Results section is also poorly organised. Also, karyotypes, and FISH experiments are mentioned but the results appeared not to be shown, and then I realised that the Figure numbers are not on the same pages as the figures, so that Figure 2 (referred to in line 179) appears to be labelled 3. Figure 4 is also referred to as 3 (also the y axes are not explained clearly). The numbering should be made clear, to avoid confusion.

*R: We apologize for the incorrect numbering of figures. In the revised text we mention the FISH experiments before we present the genomic evidence. We have also revised the legends for clarification.*

Unless there are new conclusions about the mechanism of recombination suppression, the text near line 96 should be greatly shortened.

*R: we have removed this part since it is not relevant to most results of the manuscript.*

All these, and my other negative comments, can be dealt with by revisions of the manuscript, but these changes are essential, as the present version cannot be understood without a huge effort.

*R: we have extensively revised the manuscript. We thank the reviewer’s the helpful comments and suggestions.*

I have annotated the pdf file to correct the English (red text) and used purple highlighting and text to indicate scientific errors, need for more explicit information and/or references and places where the text can be shortened because it is merely saying what is well known, so that the text can simply cite a review paper, as well as repetitions.

*R: The attached comments and suggestions are very helpful to improve the manuscript. We thank the reviewer again.*

List of major comments

The INTRODUCTION

The entire Introduction essentially says only that a fully phased and chromosome-scale assembly has been achieved of the zig-zag eel genome, a species with no detectable sex chromosome heteromorphism (references are needed for the cytological information). Several things should be in this section are not, as follows:

2. What was previously known about the relationships of the species? Do the genome data reveal for the first time that it is a close- related species of the Asian swamp eel (Monopterus albus), in the same order Synbranchiformes (line 145), or do there merely confirm this?

*R: the relationships of the species were already known. Here we merely confirm that and use the whole-genome alignment data to estimate their divergence time which is a novel result. We have streamlined the corresponding Result part in the revised manuscript since most results is already known though not through genomic analyses.*

3. The information that most Percomorpha species have a diploid number of 48 (2n), should be mentioned in the Intro (not in line 150), along with the number of species that were studied to reach this conclusion. The intro should briefly outline what is meant by the Percomorpha.

The statement that other chromosome numbers have been observed in many taxa is obscure. If it refers to other taxa it is not relevant to what the authors are trying to say. However, it seems to refer to Percomorpha, as line 153 mentions 2 others with different numbers Nile tilapia (2n = 44) and big-bellied seahorse (2n = 42), which contradicts the statement above.

The main point is presumably that a haploid number of 24 chromosomes is expected for the species studied, and the genome assembly could be consistent with this number, and this latter point is all that needs saying the the Results section.

I do not accept that a haploid number of 24 can be inferred as ancestral for Percomorpha, as the assumptions are not explained, and alternative possibilities rejected. It is not clear why this inference is important. If it is, it should be done carefully and properly, and probably other fish should be mentioned, as 24 is a fairly common number. If fish chromosome numbers have been reviewed, the paper should be cited.

*R: The reviewer is right we mainly wanted to show that the genome assembly is consistent with the haploid number. Reconstructing the ancestral karyotype for the Percomorpha clade requires more sophisticated analyses which are beyond the scope of the present manuscript. In the revised manuscript, we focus more on the karyotype characterization of zig-zag eel and have rewritten this part in the Results.*

4. Very oddly, the Introduction fails to mention that the study species of fish is sequentially hermaphroditic, and that all individuals initially develop as females but half of them later develop into males. This might be expected to suggest that the species has an environmental sex-determination system, but the observation that 50% eventually develop into males (a reference to a paper that provides proper information is needed here) might instead suggest a genetic system, and this has been confirmed by RAD seq, which found male-specific genetic markers.

*R: The detailed characterization of sequential hermaphroditism of zig-zag eel is the focus of another manuscript (Xue et al. submitted) that is under review in Aquaculture. (we suspect that sequential hermaphroditism may not necessarily contradict the presence of sex chromosome, because we observed that only the individuals carrying the Y chromosome can experience sex change, implying that sex change from female to male is associated with the temporal expression of Y-linked genes; but we are still collecting more time-serious RNA-seq and epigenetic data, at the moment we have not tested this hypothesis). We have briefly mentioned the unpublished study in the revised text and added a reference to it.*

5. The Intro should also mention the previous chromosomal assembly of zig-zag eel (fMasArm1.2) in ref.38, and explain clearly why a new assembly is needed.

*R: In the revised manuscript we mention the existing zig-zag eel genome in the introduction (instead of in the Results), and explain that the previous assembly was not useful to study the origin of the sex chromosome because the assembly has not resolved the haplotypes at the chromosome-scale.*

6. The Introduction should also outline HiFi sequencing, as this is a new approach and its basis, and the advantages and disadvantages, should be explained briefly.

*R: we have added a brief introduction of HiFi sequencing as well as adding the references.*

VAGUE STATEMENTS THAT SHOULD BE MADE MUCH CLEARER.

1. Line 100 states that "Newly accumulated transposable elements (TEs) in the partially sex-linked region can also trigger the formation of a new SLR by obstructing recombination, and cites ref. [11]. However, this is not an accepted view and therefore it is necessary to explain (briefly but very clearly) how this is supposed to work. It is misleading to say that "In other taxa, the mechanism of recombination of suppression is less clear" as it is not at all clear in the birds that this reference describes, and it is inaccurate to say that "it seems sometimes the recombination is gradually ceased " (meaning "sometimes there have been several distinct recombination suppression events" — not gradual cessation).

*R: we have removed this part because it is not relevant to the manuscprt, see the above comment for line 96.*

2. The results section should describe the evidence that the genome assembly is nearly gap-free. At present, this seems to be asserted, and the evidence is not clear.

*R: we added “The hap-Y genome seems to have a better quality in which all of the 24 chromosomes have a gap number of less than 10, with a mean gap number of 5.5, while in the hap-X genome the mean gap number is 6.4” to show that the assembled chromosomes have on average only 5 or 6 gaps.*

3. It is unclear what is meant by the suggestion that "highly repetitive [regions], such as centromeres, … may have important impacts on genome functions], as this statement is extremely vague (of course centromeres have important functions, but this is not worth mentioning, as it is well known. If the authors are trying to tell the reader something new, it needs to be less vague.

*R: since the functions of centromeres are well known, we have changed the sentence to “The high-quality genome of zig-zag eel is expected to have resolved some of the complex regions that are previously uncharacterized, such as centromeres that contains large arrays to tandem repeats” and added a reference.*

4. The units of y axes in Figure 5 are not always clear, and are not stated in the legend.

*R: we have revised the legend in Figure 3, stating more clearly about the units. To avoid repetition, in Figure 5 legend we direct the readers to the legend of Figure 3 because the data shown in those two figures were similar.*

LIST OF CONCLUSIONS THAT ARE NOT WELL SUPPORTED

1. I do not accept the conclusion that there are 2 evolutionary strata, both with X-Y sequence divergence around 1%, as there is no test of the statistical significance of the claimed difference between the 2 regions, or even confidence intervals on the divergence values, and no correction for possible sequence variation among X-linked sequences. If there really are 2 strata, the introns from which they are inferred need to be examined carefully for indels, and the alignments described in adequate detail that readers can feel assured that they are highly reliable, as even small mis-alignments could affect divergence values. The apparently striking difference in the top figure of 3a is in terms of something called "male SNPs" that is not defined, and cannot be assessed because numbers of SNPs (which I think is what the authors mean) depend on the alignment lengths. This is why divergence per nucleotide is necessary, not just counts. The panel showing a divergence measure (mystifyingly called "similarity") show at best small differences between the two regions that the authors highlight, and no confidence intervals are shown.

If there is no definitively younger stratum, one cannot hope to "identify the evolutionary forces that favor the recombination suppression in the SLR"

*R: During the revision, we tested whether the two strata have significant differences in the density of male-specific variants and X-Y sequence divergence. Though we did find a significant difference in X-Y sequence divergence between the two strata (Fig. 3e), the values are both close to 99%, as the reviewer pointed out. Even the two regions truly represented two evolutionary strata, they probably have a similar age of origin; but classic evolutionary strata refer to regions with large differences. So to avoid confusion, we name these two regions simply as “two regions” instead of “two strata”. And we write “It is possible that R1 and R2 represent two evolutionary strata, but we cannot rule out the possibility that there is only one stratum but one region diverges faster than the other, similar to the scenario reported in emu”. And to clarify, “male SNP” refers to the density of male-specific SNPs, and we have accordingly changed “male SNP” to “SNP density” and gave a clear description about the unit in the legend.*

2. The statement that recent studies on ratites suggested that a change of chromatin configuration and recombination rate can occur before sequence differentiation of the sex chromosomes is not convincing evidence for the idea that the presence of heterochromatin is a cause of suppressed recombination. It is well understood that suppressed recombination leads quickly to repetitive sequence accumulation (and evolution of heterochromatin) and that sequence divergence is much slower. Therefore, these observations are perfectly compatible with the order (i) recombination suppression (for whatever reason), (ii) evolution of heterochromatin, (iii) sequence divergence.

*R: we agree with the reviewer that it is not straightforward to determine which events came prior to the others in the case of emu and other ratites. Some other systems may provide a clearer picture of the relationships between recombination suppression and heterochromatinization. For now, we have removed the discussion about the studies in ratites which may not be proper evidence to support the hypothesis that heterochromatin induces recombination suppression. We apologize for our uncareful interpretations of the ratite study and thank the reviewer for pointing this out.*

Reviewer #2

In this manuscript the near gap free assembly of the zig-zag eel is described. Importantly in this study most centromere and subtelomere where sequenced on most chromosomes. The main focus of the paper is the analysis of the sex chromosomes. In depth sequencing of X and Y chromosomes supports the authors main conclusions that pericentromeric heterochromatin may have existed on the photo sex chromosome leading to low recombination and establishment of sexually antagonistic /sex determination genes. While the data are sound and conclusions interesting for the field I have some concerns with the presentation of the work.

*R: We appreciate the reviewer’s positive remarks on the manuscript.*

Generally the paper will benefit from some English editing, I spotted a number of spelling or grammar errors some listed below.

*R: In the new version we have edited the English language throughout the manuscript.*

Also the introduction has much information about sex chromosomes mostly in reptiles and mammals. There is also a lot of information about the sequencing strategy but I feel there is lack of information about the eel itself, how does it fit in the diversity of fish (in terms of genomes and sex chromosome systems in general). What is known about their sex determination and sexual development? Some of this information is provided in the results section. It was unclear to me if the male specific markers had been mapped to the putative XY?

*R: Thanks for the comments that we believe can improve the writing of the Introduction part. in the revised manuscript we introduced the zig-zag eel in the Introduction part instead of in the Results, and described previous and one (currently) unpublished study on zig-zag eel sex determination. The reason we chose to study this fish is that it is newly understood to have a sex chromosome pair though it is a hermaphroditic fish like the Asian swamp eel. So we propose this fish may represent a species in the transition from hermaphroditism to dioecism though we are still collecting more time-series RNA-seq and epigenetic data to confirm this. We also mention the available zig-zag eel genome, and explained why we needed a new diploid genome assembly that contains separate X and Y chromosomes. We have actually confirmed that the previously identified male-specific markers on the sex-linked region on the Y chromosome, and mentioned this result in the first part of the Result in line 288-290 and added a supplementary figure (Fig. S2).*

The section on the identification and strata analysis is very interesting. As for the strata analysis and claim that they are young sex chromosomes I was wondering how this was established. I know many fish sex chromosomes are homomorphic but are they all "young" and is this because we apply assumptions from other species. In fish sex determination and sexual development is more dynamic and sex chromosomes and sex linked genes may have different functions) shaping their evolutionary trajectory in different ways.

*R: It’s true that sex chromosomes differ from those of other systems in terms of the extent of degeneration. As in the case of zig-zag eel, the sex chromosome pair is not differentiated though we do not know the exact time when they originated. But there are fish species that have homomorphic sex chromosomes but have ancient origins. It is still a matter of debate why fish sex chromosomes are usually homomorphic, but there are a few hypotheses proposed, for instance some fish can occasionally experience sex reversal so that preventing sex chromosomes from degeneration, or the XY chromosomes can occasionally recombine; turnover of sex chromosomes is also frequent in fish so the sex chromosomes have recent origins; some authors argue that homomorphic sex chromosome should be the “norm” while the mammalian and avian sex chromosomes are exceptional.*

*To identify the sex-linked region in the relatively young sex chromosomes of zig-zag eel, our approach was to identify sex-linked variants using population genetics methods - we identified male-specific SNPs that were restricted to a ~7 Mb region that was considered as the sex-linked region. However, given the patterns of two evolutionary strata are not sufficiently clear, in the revised manuscript we refer to the two distinct sequences simply as “two regions” instead of “two evolutionary strata”.*

Line 230: I would suggest to find a better title.

*R: the title was changed from “extended pericentromeric heterochromatin” to “broad pericentromeric heterochromatin domains”.*

238: please remind the reader what PCH is.

*R: we use the “pericentromeric region” instead.*

There are a number of topics that the discussion touches on which are very interesting. I would have like more discussion on the other genes identified in this non recombining region and it they have a wide expression patter as has recently be described for mammalian sex chromosomes?

*R: During this revision we compared the dosage sensitivity and tissue-specificity of the genes in and outside the sex-linked regions. We found the genes in the SLR do not seem to differ from those in the other part of the genome in terms of how broadly they are expressed, though they seem to be more sensitive in dosage. These results are shown in the supplementary figure S8, and we showed the full gene list as well as gene expression levels across tissues in the supplementary table S3.*

line 45/46 revise

*R: we have removed this sentence since we fear that our data does not perfectly support the sexual antagonistic polymorphism hypothesis. Instead, we focus more on the origin of the sex-linked region from the pericentromere.*

line 56 revise sentence

*R: we have revised the sentence to “The sex chromosomes originated from an ordinary autosome pair. Theoretical studies predict that recombination suppression in the sex-linked region (SLR) is selected when sexually antagonistic polymorphism establishes close to the sex-determining locus”.*

131: what are the pseudo-chromosomes

*R: to avoid confusion, we now refer to them as chromosomes or chromosomal assemblies.*

165: found that first sentence a bit confusing.

*R: we have revised the sentence to “The high-quality genome of zig-zag eel is expected to have resolved some of the complex regions that are previously uncharacterized, such as centromeres”.*

271: revise sentence

*R: we have revised the sentence to “we speculate that the divergence between the X- and Y-linked region, although low, allows clearer partitioning of haploid-specific reads that help resolve repetitive regions”*

The work represented in this manuscript is substantial and the in depth analysis specifically of the sex chromosomes is interesting. I felt that there was a lot of other interesting aspects that the authors did not discuss in much detail as they chose to focus on the sex chromosomes. However even on sex chromosomes there is little discussion and presentation e.g. of the genes in the sex specific region.

*R: many thanks for the positive remarks. Since the X and Y chromosome do not actually differ in gene content, we did not focus on the discussions about the general pattern of those sex-linked genes. We have now compared the dosage sensitivity and tissue-specificity of the genes in and outside the sex-linked regions and show the result in the supplementary Fig. S8 and show the gene list in the supplementary table S3. In this revision, we paid more attention to the candidate sex-determining gene HMGN6 and used more vertebrate species to illustrate the origin of this gene in zig-zag eel (and teleost in general). We thank the reviewer again for the time reviewing our manuscript.*

**Second round of review**

**Reviewer 1**

This ms is improved, but still needs work. I have noted some comments on the pdf file, and indicated some further changes including deleting some words and replacing them with others. I hope that the authors will be able to see these.

I have a few further comments.

1. It is not explained how one can “identify the evolutionary forces that favor the recombination suppression in the SLR” by examining “the expression profiles of genes in the SLR in the gonads of males and females as well as the intersex individuals”. If these data cannot identify these evolutionary forces (as I think they cannot), this claim should not be made.

It would be more accurate simply to say that the study “examined the expression profiles …. which did not reveal any tissue-specific expression”. On the other hand, contradicting this statement about the overall picture (which seems confusing unless there is some reason for expecting these genes to show some overall tissue expression pattern), SYCE3 is expressed only in testis and Sertoli cells in the late stage of ovotestis tissue, suggesting involvement in spermatogenesis and in the mature testis, consistent with its known role in meiosis [43]; similarly (though for some reason the text says “In contrast”), HMGN6 a gene with a High Mobility Group domain (which is also found in the mammalian master sex-determining gene Sry, though HMGN6 is known only in teleosts) shows higher expression in testis and early stages of ovotestis at a similar level, than in ovaries.

2. In the same results section, it is claimed that the SLR genes “are more dosage-sensitive compared to genes in the other part of the genome”, but again this is not explained and the text merely refers to a Supplementary file. How is dosage sensitivity defined, and why might it be interesting?

3. PCH in line 236 is not defined.

4. Line 224 says that both SYCE3 and HMGN6 are located in the S1, but the term S1 has not been explained.

5. It is stated in line 233 that the pericentromeric regions have (as expected) “lower recombination rates”, and the Methods section includes a section about recombination rate estimations. However, this simply says that the study used the ReLERNN (1.0.0) [84] method, but does not even mention the principle on which this method is based. Presumably, as it uses re-sequenced genomes, it employs linkage disequilibrium to infer regions of higher and lower recombination rates. The ms should make this clear. At present, readers will believe that recombination rates have been estimated. To actually estimate recombination rates from LD data (shown at the top of Figure 5a) one must make several assumptions, and the ms should provide an explanation of these, including values of any parameters assumed. Some statistics should also be provided, such as confidence intervals for the values. As shown, readers don’t know whether there is enough reliable information in the low recombination regions, or not.

It also seems odd that the lower figures in 5a include these recombination rate estimates, but the similar figures for the sex chromosome pair (Fig 3) do not.

6. The Discussion section needs further shortening, to make the conclusions clearer, and there is no need to repeat these again in a separate Conclusions section, which should be deleted. I have suggested these on the pdf file. Specifically (among other points), the revised version now mentions that this fish is an example where the sex-determining locus appears to be located within a pericentromeric region with infrequent recombination. However, the discussion about this is inadequate. It is not clear whether the presence of the sex-determining locus within such a region reflects a selective process that favored such a non-recombining region, or whether the recent appearance of this locus in such a region occurred by chance, given that these regions occupy substantial proportions of this fish’s genome.

7. The statement that evidence that HMGN6 is likely an ortholog of HMGN5 that is present in other vertebrates should remind readers of where this evidence is shown in the paper. I was unable to find this result.

**Reviewer 2**

**Authors Response**

**Point-by-point responses to the reviewers’ comments:**

Reviewer #1: This ms is improved, but still needs work. I have noted some comments on the pdf file, and indicated some further changes including deleting some words and replacing them with others. I hope that the authors will be able to see these.

*R: Thank you again for reviewing our manuscript. We have access to the pdf file, and it (like the previous one) is very helpful to improve the writing and clarity of the manuscript.*

I have a few further comments.

1. It is not explained how one can “identify the evolutionary forces that favor the recombination suppression in the SLR” by examining “the expression profiles of genes in the SLR in the gonads of males and females as well as the intersex individuals”. If these data cannot identify these evolutionary forces (as I think they cannot), this claim should not be made.

It would be more accurate simply to say that the study “examined the expression profiles …. which did not reveal any tissue-specific expression”. On the other hand, contradicting this statement about the overall picture (which seems confusing unless there is some reason for expecting these genes to show some overall tissue expression pattern), SYCE3 is expressed only in testis and Sertoli cells in the late stage of ovotestis tissue, suggesting involvement in spermatogenesis and in the mature testis, consistent with its known role in meiosis [43]; similarly (though for some reason the text says “In contrast”), HMGN6 a gene with a High Mobility Group domain (which is also found in the mammalian master sex-determining gene Sry, though HMGN6 is known only in teleosts) shows higher expression in testis and early stages of ovotestis at a similar level, than in ovaries.

*R: We thank the reviewer for raising this issue. We realize that our RNA-seq data cannot “identify the evolutionary forces that favor the recombination suppression in the SLR” as we hoped. We have now removed this sentence and directly described our results as the reviewer suggested.*

*We believe that the majority of the SLR genes should behave similarly to autosomal genes, because the sex chromosomes are young and have not specialized. However, those involved in sex determination and sexual differentiation are expected to show testis- or ovary-biased expression. This is the reason we focused on SYCE3 and HMGN6 that showed signals of testis-limited expression. But we also found a disparity of these two genes: the cellular locations and timing of expression indicated that HMGN6 is likely linked to sex determination while SYCE3 is involved in spermatogenesis thus not a sex-determining gene.*

2. In the same results section, it is claimed that the SLR genes “are more dosage-sensitive compared to genes in the other part of the genome”, but again this is not explained and the text merely refers to a Supplementary file. How is dosage sensitivity defined, and why might it be interesting?

*R: Previous studies in birds and snakes suggest that genes in the degenerated sex-limited chromosome tend to be dosage-sensitive. However, given that in zig-zag eel the sex chromosomes barely degenerate, we now realize that it is not needed to compare the dosage sensitivity between SLR genes and autosomal genes. We have thus removed the result about dosage sensitivity.*

3. PCH in line 236 is not defined.

*R: We have defined PCH (pericentromeric heterochromatin) in the previous section. To avoid repetition, we did not define it here again.*

4. Line 224 says that both SYCE3 and HMGN6 are located in the S1, but the term S1 has not been explained.

*R: We apologize for the typo: it should be “R1”. We have now corrected this typo.*

5. It is stated in line 233 that the pericentromeric regions have (as expected) “lower recombination rates”, and the Methods section includes a section about recombination rate estimations. However, this simply says that the study used the ReLERNN (1.0.0) [84] method, but does not even mention the principle on which this method is based. Presumably, as it uses re-sequenced genomes, it employs linkage disequilibrium to infer regions of higher and lower recombination rates. The ms should make this clear. At present, readers will believe that recombination rates have been estimated. To actually estimate recombination rates from LD data (shown at the top of Figure 5a) one must make several assumptions, and the ms should provide an explanation of these, including values of any parameters assumed. Some statistics should also be provided, such as confidence intervals for the values. As shown, readers don’t know whether there is enough reliable information in the low recombination

regions, or not.

It also seems odd that the lower figures in 5a include these recombination rate estimates, but the similar figures for the sex chromosome pair (Fig 3) do not.

*R: we have indeed used 20 individually re-sequenced genomes to estimate the recombination rate. The ReLERNN program uses a machine learning approach (Adrion et al. 2020 MBE) to estimate the recombination rate instead of using linkage disequilibrium. We provide a bit more information in the Methods section, as well as a supplementary table (Table S5) to show the raw result of the ReLERNN program. Because the ReLERNN program presumes the genome to be diploid, we could not use it to estimate the recombination rate for the sex chromosomes which are haploid (but not for the pseudoautosomal region). We explained this in the Methods part.*

6. The Discussion section needs further shortening, to make the conclusions clearer, and there is no need to repeat these again in a separate Conclusions section, which should be deleted. I have suggested these on the pdf file. Specifically (among other points), the revised version now mentions that this fish is an example where the sex-determining locus appears to be located within a pericentromeric region with infrequent recombination. However, the discussion about this is inadequate. It is not clear whether the presence of the sex-determining locus within such a region reflects a selective process that favored such a non-recombining region, or whether the recent appearance of this locus in such a region occurred by chance, given that these regions occupy substantial proportions of this fish’s genome.

*R: We have revised the Discussion section to make it more concise by taking the suggestions from the reviewer. The reviewer also raised a very good point about whether the appearance of the sex-determining allele in the pericentromeric region is simply by chance. In the revised text we added: “Because the lowly recombining pericentromeric regions are large, occupying substantial proportions of the genome, it’s possible that the origin of sex-determining allele occurred in such regions by chance. We note, however, the vast majority of the pericentromeric regions is gene-poor, and the candidate sex-determining gene HMGN6 is very close to the centromere where recombination should be very rare. We also cannot rule out the possibility that the sex-determining gene may have been translocated into the low-recombination region. This hypothesis cannot be tested without high-quality genomes of closely-related species”*

7. The statement that evidence that HMGN6 is likely an ortholog of HMGN5 that is present in other vertebrates should remind readers of where this evidence is shown in the paper. I was unable to find this result.

*R: we have uploaded the supplementary tables and figures (like in the previous submission) which should be able to be downloaded. We hope the reviewer can find it this time, and thank again for the helpful reviews.*
